# Simple Cobalt Nanoparticle-Catalyzed Reductive Amination for Selective Synthesis of a Broad Range of Primary Amines

**DOI:** 10.3390/molecules30153089

**Published:** 2025-07-23

**Authors:** Bingxiao Zheng, Liqin Yang, Yashuang Hei, Ling Yu, Sisi Wen, Lisi Ba, Long Ao, Zhiju Zhao

**Affiliations:** Functional Polymer Materials R&D and Engineering Application Technology Innovation Center of Hebei Province, Xingtai University, Xingtai 054001, China; yanglq2005@163.com (L.Y.); heiys0815@163.com (Y.H.); 200820347@xttc.edu.cn (L.Y.); 18844115900@163.com (S.W.); balisi814@163.com (L.B.); 18131425341@163.com (L.A.)

**Keywords:** simple cobalt nanoparticles, earth-abundant metal, carbonyl compounds, reductive amination, primary amines

## Abstract

In the field of green chemistry, the development of more sustainable and cost-efficient methods for synthesizing primary amines is of paramount importance, with catalyst research being central to this effort. This work presents a facile, aqueous-phase synthesis of highly active cobalt catalysts (Co-Ph@SiO_2_(x)) via pyrolysis of silica-supported cobalt–phenanthroline complexes. The optimized Co-Ph@SiO_2_(900) catalyst achieved exceptional performance (>99% conversion, >98% selectivity) in the reductive amination of acetophenone to 1-phenylethanamine using NH_3_/H_2_. Systematic studies revealed that its exceptional performance originates from the in situ pyrolysis of the cobalt–phyllosilicate complex. This process promotes the uniform distribution of metal cobalt nanoparticles, simultaneously enhancing porosity and imparting bifunctional (acidic and basic) properties to the catalyst, resulting in outstanding catalytic activity and selectivity. The catalyst demonstrated broad applicability, efficiently converting diverse ketones (aryl-alkyl, dialkyl, bioactive) and aldehydes (halogenated, heterocyclic, biomass-derived) into primary amines with high yields (up to 99%) and chemoselectivity (>40 examples). This sustainable, non-noble metal-based catalyst system offers significant potential for industrial primary amine synthesis and provides a versatile tool for developing highly selective and active heterogeneous catalysts.

## 1. Introduction

Primary amines serve as essential precursors for fine chemicals, pharmaceuticals, agrochemicals, and advanced materials [1,2,3]. Key strategies for preparing primary amines encompass N-alkylation of ammonia using haloalkanes or alcohols [4,5,6], reduction of specific nitrogen-containing compounds [7,8,9,10], and reductive amination of carbonyl compounds [11,12,13,14]. The synthesis of primary amines from carbonyl compounds, NH_3_, and H_2_ is particularly significant due to the low cost and wide availability of these feedstocks [14]. However, selectivity control remains challenging, with competing side reactions (e.g., over-alkylation and carbonyl reduction to alcohols) limiting efficiency [15,16]. Developing robust catalysts to address these issues is therefore a critical technological objective.

Conventional catalysts for this transformation primarily rely on precious metals (Ru, Ir, Pt, Rh, Pd) in heterogeneous or homogeneous forms [14,17,18,19,20,21,22], alongside some Ni-based homogeneous complexes [23]. While effective, homogeneous systems face limitations in recyclability, and noble metals hinder large-scale applications due to cost. Consequently, research focuses on designing heterogeneous non-noble metal catalysts (Co, Ni, Fe, Cu) [12,24,25,26,27,28,29,30,31,32,33] that combine high activity, selectivity, and cost efficiency for reductive amination.

Currently, Co-, Ni- and Fe-based heterogeneous materials with high selectivity and activity were obtained by pyrolysis of organometallic complexes or metal–organic frameworks on heterogeneous supports for reductive amination of carbonyl compounds [12,24,25,26,27,28]. But the reported heterogeneous 3d metal-based catalytic systems also demonstrated some drawbacks including cumbersome synthesis steps (three steps or more), harsh synthesis conditions (150 °C; >24 h), the use of organic solvents (N,N-dimethylformamide, acetonitrile or methanol) and specific equipment (Teflon-lined stainless-steel autoclaves) in the synthesis process of precursors [12,24,25,26,27,28]. Although these preparations represent highly useful tools to produce novel nano-structured catalysts on a lab scale, the upscaling can be difficult and requires specialized equipment. Therefore, the development of a simple and green synthesis process for catalysts is of great significance for the industrialization of the synthesis of primary amines.

Herein, we have developed a series of cobalt-based catalysts by in situ growth for reductive amination reactions using NH_3_ and molecular H_2_ to produce primary amines. In comparison with the already disclosed synthesis methodologies, this novel catalytic system would hold multiple advantages, including simplicity (one-pot synthesis under mild conditions), cost-effectiveness (using nonprecious metals as catalyst), sustainability (water-based solvent system in the preparation process), practicality (facile magnetic separation of the catalyst), and versatility (broad precursor compatibility). Moreover, the synergistic cooperation between the active metal species and the ligand resulted in its high activity, and the wide applicability of this catalyst to other substrates with other reducible groups further confirms its great potential.

## 2. Results and Discussion

### 2.1. Preparation of the Co-Ph@SiO_2_(x)

**Figure 1:**
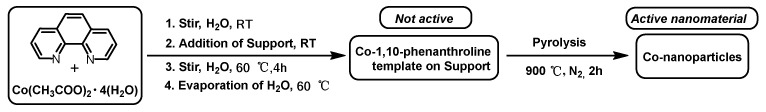
Preparation of cobalt nanoparticles supported on silica or other support by the pyrolysis of cobalt–phenanthroline complex.

To prepare supported Co nanoparticles, Co–phenanthroline was first immobilized on silica. This was achieved by stirring a mixture of Co(OAc)_2_•4H_2_O, 1,10-phenanthroline, and silica in water at 60 °C, followed by slow solvent evaporation. The resulting Co–phenanthroline complex formed a stable precursor on the silica support (Figure 1). Subsequently, this templated solid compound was pyrolyzed at temperatures ranging from 700 °C to 1000 °C under nitrogen atmosphere (see Supporting Information) [34]. The resulting Co materials are designated as Co-Ph@SiO_2_(x), where Ph denotes 1,10-phenanthroline and x represents the pyrolysis temperature.

### 2.2. Catalytic Performance of Various Catalysts

The reductive amination of acetophenone to 1-phenylethanamine was selected as a model reaction to evaluate catalysts for primary amine synthesis under defined conditions (Table 1). Control experiments confirmed the necessity of catalysis: no target amine was detected without a catalyst (Table 1, entry 1), and the Co–phenanthroline precursor on silica showed no imine conversion, indicating inactive cobalt species (Table 1, entry 2). Strikingly, pyrolysis (700–1000 °C) of this precursor generated highly active materials (Table 1, entries 3–7). Catalytic performance exhibited significant pyrolysis temperature dependence: Co-Ph@SiO_2_(700) gave 71.4% acetophenone conversion with selectivities of 36.2% (1-phenylethanamine), 27.7% (imine), and 35.0% (Schiff base) (Table 1, entry 3). Performance improved substantially at 800 °C (57.3% yield, 69.4% selectivity to 1-phenylethanamine; Table 1, entry 4), peaking at 900 °C with 92.1% conversion and 82.2% selectivity (Table 1, entry 5). However, increasing pyrolysis temperature to 1000 °C drastically reduced conversion (65.1%) and primary amine selectivity (2.2%), likely due to cobalt agglomeration and C-N structure degradation (Table 1, entry 6; Figure S2). Notably, optimized conditions with Co-Ph@SiO_2_(900) achieved near-quantitative performance (>99% conversion, >98% selectivity; Table 1, entry 7).

Catalyst versatility was further investigated using alternative supports (C, Al_2_O_3_, ZrO_2_, TiO_2_, CeO_2_, Nb_2_O_5_; Table S1). All cobalt–phenanthroline catalysts exhibited high activity (>93% 1-phenylethanamine yield), demonstrating broad support adaptability. In contrast, analogous Ni-Ph@SiO_2_(900) and Fe-Ph@SiO_2_(900) catalysts were completely inactive (Table 1, entries 8–9). These results unequivocally establish Co-Ph@SiO_2_(900) as the optimal catalyst under standard conditions for acetophenone reductive amination. Consequently, this catalyst was employed for subsequent reaction optimization studies.

### 2.3. Reasons for the High Activity of Co-Ph@SiO2(900)

**Figure 2:**
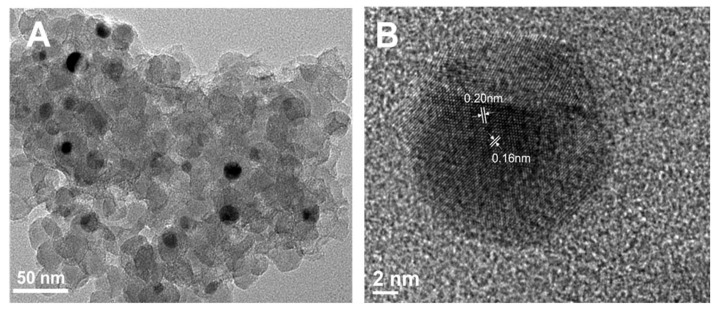
(**A**) Low-magnification HAADF-STEM image of Co-Ph@SiO_2_(900); (**B**) zoom-in HAADFSTEM image of encapsulated Co nanoparticles in Co-Ph@SiO_2_(900).

To elucidate the structural features and catalytic role of the cobalt–phenanthroline complex, characterization was performed using SEM, TEM, BET, XPS, and TPD. SEM analysis (Figure S1) confirmed the silica carrier provides uniform structural support. TEM imaging (Figure 2A) revealed uniformly dispersed cobalt nanoparticles. EDS elemental mapping further demonstrated homogeneous distributions of C, N, O, Co, and Si throughout the Co-Ph@SiO_2_(x) materials (Figure S4). While pyrolysis temperature increases from 700 °C to 900 °C induced a gradual cobalt particle size increase (Figures S2a–c), negligible aggregation was observed. In stark contrast, Co-Ph@SiO_2_(1000) exhibited severe cobalt particle aggregation, with diameters sharply increasing to ~50 nm (Figure S2d). HR-TEM analysis of the optimal Co-Ph@SiO_2_(900) catalyst (Figure 2B) revealed crystalline cobalt nanoparticles tightly encapsulated by a graphitized carbon layer. These particles displayed distinct lattice fringes with spacings of 2.05 Å and 1.79 Å, corresponding to the (111) and (200) planes of fcc cobalt, respectively. This encapsulating carbon layer critically prevented particle aggregation at temperatures ≤ 900 °C. However, its protective function was compromised at 1000 °C, directly responsible for the severe aggregation observed.

**Figure 3:**
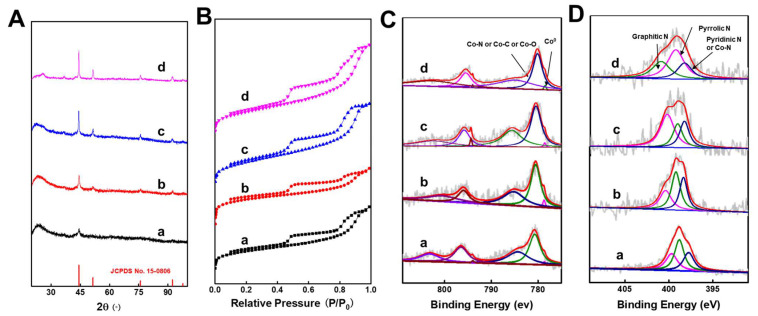
(**A**) Powder XRD patterns, (**B**) N_2_ adsorption–desorption isotherms, (**C**) Co 2p XPS spectra and (**D**) N 1s XPS spectra. In the figures, a, b, c, and d represent Co-Ph@SiO2(700), Co-Ph@SiO2(800), Co-Ph@SiO2(900), and Co-Ph@SiO2(1000), respectively.

XRD analysis of Co-Ph@SiO_2_(x) catalysts (Figure 3A) confirmed metallic cobalt formation, exhibiting characteristic diffraction peaks at 44.2°, 51.6°, 76.0°, 92.4°, and 97.8° (JCPDS 15-0806). Crystallinity progressively increased with pyrolysis temperature from 700 °C to 1000 °C. Nitrogen physisorption at 77 K (Figure 3B) revealed type-I microporous structures, with BET surface areas ranging within 80–150 m^2^ g^−1^ (Table S3). Crucially, Co-Ph@SiO_2_(900) demonstrated 128.12 m^2^ g^−1^ surface area versus 62.62 m^2^ g^−1^ for ligand-free Co@SiO_2_(900) (Table S2), evidencing the phenanthroline ligand’s role in enhancing porosity—a key factor for superior catalytic performance (Table 1, entries 7 and 10). XPS survey spectra confirmed surface presence of Co, C, N, O, and Si (Figure S5). The Co 2p spectrum showed that there were three Co species in all the four Co-Ph@SiO_2_(x) samples, including metallic Co^0^ at 778.5 eV and Co-O/Co-N/Co-C at 780.5 eV (Figure 3C). The presence of Co^0^ (778.5 eV) in Co-Ph@SiO_2_(x) implied the partial reduction of cobalt during the pyrolysis process, which was consistent with the results of XRD examinations (Figure 3A). Deconvoluted N 1s spectra (Figure 3D) resolved four chemical environments: pyridinic/Co-N (398.1 eV), pyrrolic (399.1 eV), and graphitic (400.6 eV). Notably, graphitic and oxidized nitrogen content increased with pyrolysis temperature, while target product yield exhibited an inverse relationship—initially rising then declining above 900 °C. This performance inversion correlates directly with high-temperature cobalt agglomeration and nitrogen species volatilization, as established by TEM analysis.

Notably, Co-Ph@SiO_2_(900) exhibits significantly greater acidity than Co@SiO_2_(900) (Figure S6). This enhanced acidity facilitates C=N group activation in in situ-generated imines and Schiff bases through acid site interactions with nitrogen atoms in C=N bonds (Figure S7), thereby promoting reaction progression. CO_2_-TPD analysis (Figure S8) confirms Co-Ph@SiO_2_(900) possesses substantially more numerous and stronger basic sites compared to Co@SiO_2_(900) [35,36]. These observations collectively indicate metallic cobalt primarily interacts with basic sites in Co-Ph@SiO_2_(900), resulting in higher electron density at cobalt centers relative to Co@SiO_2_(900). The elevated electron density enhances formation of active hydrogen species on cobalt surfaces (Figure S9), facilitating hydrogenation of imines and Schiff bases. Elemental analysis reveals cobalt contents of 4–6 wt% across composites (Table S1). ICP-OES quantification demonstrates increasing cobalt content in Co-Ph@SiO_2_(x) with pyrolysis temperature (Table S2), consistent with progressive decomposition of organic components. Collectively, these characterization data establish that phenanthroline incorporation profoundly modifies cobalt nanoparticle characteristics—including electronic nature, size distribution, and morphological features—thereby governing catalytic functionality.

### 2.4. Optimization of Reaction Conditions

**Figure 4:**
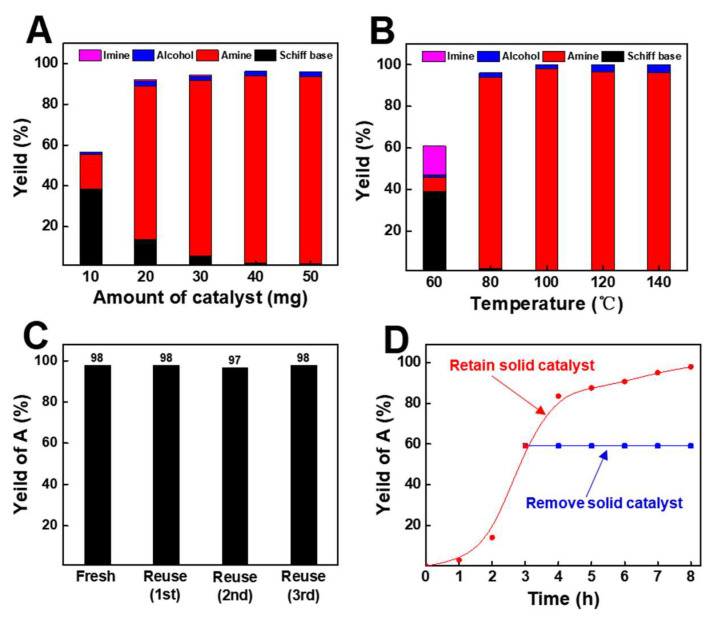
(**A**) Effect of amount of catalyst; (**B**) effect of different temperatures; (**C**) reusability of Co-Ph@SiO_2_(900); (**D**) time–yield plots for reductive amination of acetophenone Co-Ph@SiO_2_(900) (red line) and removing Co-Ph@SiO_2_(900) after 3 h (Blue line).

Generally, the amount of catalyst can significantly affect the distribution of products and conversion of raw material. When the amount of Co-Ph@SiO_2_(900) increased from 10 to 50 mg (Figure 4A), and the Co usage affected both the distribution and conversion of products. Too-low usage of Co (<40 mg) was unbeneficial for the formation of 1-phenylethanamine owing to the insufficient active sites. When the usage of catalyst was 50 mg, the selectivity and yield of 1-phenethylamine remained constant with the previous conditions (40 mg). From the viewpoint of achieving high selectivity of 1-phenylethanamine at a suitable reactivity, 40 mg was selected as the optimal usage of catalyst [12,13].

Subsequently, the reaction temperature can significantly affect the catalytic activity and selectivity. It was observed that the conversion of acetophenone increased with the sharply increase in temperature from 60 to 80 °C (Figure 4B). In comparison, the selectivity of 1-phenylethanamine was increased at temperatures from 60 to 100 °C and then decreased when the temperature further increased from 100 to 140 °C. It is mainly because more 1-phenylethanol would be generated from the direct hydrogenation of acetophenone with high temperature.

Additionally, as important properties of a heterogeneous catalyst, the durability of Co-Ph@SiO_2_(900) was evaluated. Co-Ph@SiO_2_(900) could be recycled at least four times without a decline in the catalytic activity and product selectivity (Figure 4C). After the reaction, a magnet was placed adjacent to the reactor wall, rapidly attracting all powders to the wall (Figure S10). This demonstrates the strong magnetic properties of the Co-based material. Thus, the catalyst could be easily separated from the reaction mixture just by pouring the solution. Moreover, no obvious change was observed between the virgin and recovered Co-Ph@SiO_2_(900) as characterized by SEM, TEM, XPS and XRD (Figures S11 and S12), further indicating the high durability of Co-Ph@SiO_2_(900). Additionally, the yield of 1-phenylethanamine plateaued after Co-Ph@SiO_2_(900) was removed from the reaction system after 3 h (Figure 4D), and no Co species were detected by ICP examination (Table S2). These results firmly verified the heterogeneous nature of Co-Ph@SiO_2_(900).

### 2.5. Substrate Scope

Delighted by the excellent result of the reductive amination of acetophenone, the scope of the substrates (both ketones and aldehydes) was explored to show the applicability of this strategy to synthesize primary amines (Table 2, Table 3 and Table 4). First, we investigated the reductive amination of acetophenones with various substituents. Substrates with long aliphatic moiety (Table 2, entries 1 and 2) could be obtained in very good yields of 95–99%. The conversion of acetophenones with electron-withdrawing substituents, such as halogens, worked out well with yields of over 95% for the para-substituted derivatives (Table 2, entries 3 and 4). The sterically more demanding meta- and ortho-substituted derivatives (Table 2, entries 5–7) could also be converted with yield of >95%. The heavily electron-withdrawing trifluoromethyl group (Table 2, entry 8) required a long reaction time (24 h) to be converted into its corresponding primary amine with an excellent yield of 98%. Minor electron-donating substituents, such as methyl and ethyl groups, could be tolerated very well in meta- or para-positions of the aromatic ring (Table 2, entries 9–11). For the ortho-substituted acetophenone, we had to increase the reaction time to 12 h to obtain 96% of isolated product (Table 2, entry 12). If the electron-donating property of the substituents was increased (e. g., methoxy groups), meta- and para-substituted ketones (Table 2, entries 13 and 14) could be converted smoothly into the corresponding primary amines without any need to increase the temperature. Meanwhile, Co-Ph@SiO_2_(900) has a higher TOF compared with other reported earth-abundant metal-based catalytic systems (Table S4).

Second, delighted by the good result for the reductive amination of acetophenones, the scope of reductive amination of other ketones was investigated to show the applicability of this strategy to synthesize primary amines over Co-Ph@SiO_2_(900) (Table 3). In our catalytic system, all examined alkyl ketones (Table 3, entries 1–8) gave good yields and even 2-adamantanone and androsterone (a bioactive molecule) with a complex spatial structure could be efficiently converted into the corresponding primary amines (Table 3, entries 9 and 10).

Moreover, in comparison with ketones, we further investigated the more challenging aldehyde compounds. It is very important to achieve a high degree of chemoselectivity for organic synthesis and drug discovery. Obtaining primary amines with high selectivity is more challenging because of their higher reactivity. Surprisingly, Co-Ph@SiO_2_(900) showed a high activity for reductive amination of various carbonyl compounds (Table 4), and the corresponding primary amines could be obtained with high selectivity. In this regard, we conducted the reaction of sterically hindered (Table 4, entries 1–4), sensitive halogenated (Table 4, entries 5–7) and functionalized benzaldehydes (Table 4, entries 8–18). Delightfully, alkyl aldehydes and aryl aldehydes with reduction-sensitive substituents could all be converted into the desired primary amines with high yields, and those groups remained intact. In addition, 5-hydroxymethylfurfural (a biomass platform molecule) and citronellal (a plant molecule) could also be transformed into the corresponding primary amines with high yields of 73% and 95% respectively (Table 4, entries 17–18). It further proved the great potential of the catalytic system in reductive amination for selective synthesis of a broad range of primary amines.

### 2.6. Mechanism Investigation

**Figure 5:**
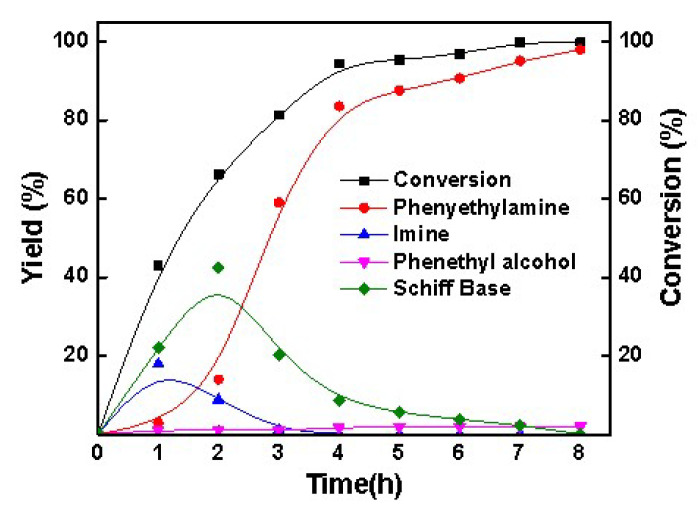
Time–yield plots. Reaction conditions: acetophenones, 1 mmol; methanol, 3 mL; H_2_, 3.4 MPa; NH_3_, 0.6 MPa; Co-Ph@SiO_2_(900), 40 mg; 100 °C; 12 h.

To investigate the reaction cascade mechanism for the Co-Ph@SiO_2_(900)-catalyzed reductive amination of the carbonyl compounds, the dependence of the product distribution on time was studied in detail by employing reductive amination of acetophenone as an example (Figure 5). As shown in Figure 5, most of the acetophenone was rapidly consumed in 2 h to form imine and Schiff base. Little of 1-phenylethanamine was detected in the 2 h because the formed 1-phenylethanamine from imine hydrogenation could easily react with acetophenone to generate Schiff base (Figure S13a,b). 1-phenethylamine was rapidly formed when most of the acetophenone was converted within 2–4 h. In the reaction process, nearly no 1-phenylethanol was detected because the imine formed much more quickly, which resulted in the rapid conversion of acetophenone with NH_3_ and thus decreased the direct hydrogenation of acetophenone to a large extent. And another reason is that the Co-Ph@SiO_2_(900) had a weak hydrogenation ability of acetophenone to 1-phenylethanol at 100 °C (Figure S13c). Moreover, control experiments using Schiff base as the reactant over Co-Ph@SiO_2_(900) (Figure S13d,e) indicated that the primary amine was yielded in the presence of NH_3_, while the secondary amine was formed without NH_3_. These results further clearly explained the absence of secondary amine in the entire reaction process. Additionally, when using aldehydes as the substrates, the formed imines would be converted into imidazolines (Figure S14) at a very low yield.

**Figure 6:**
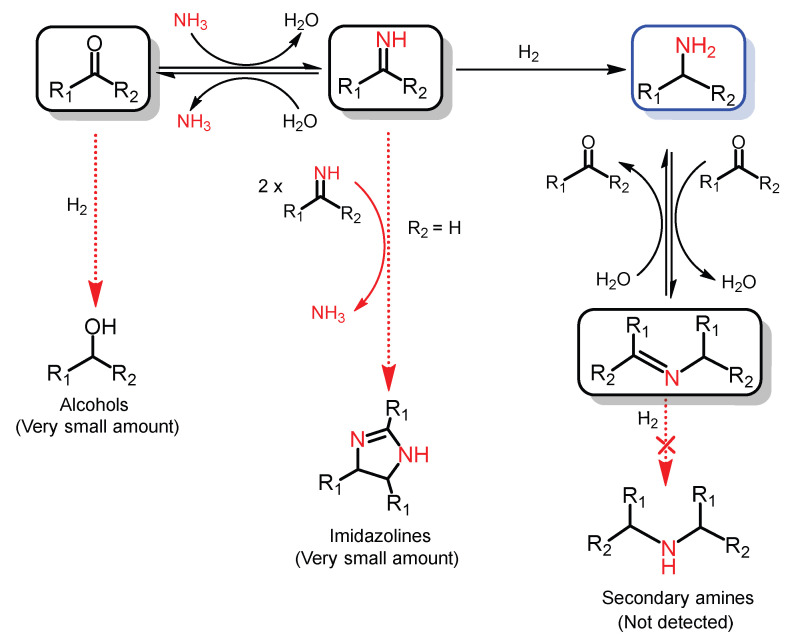
Proposed mechanism for the reductive amination of carbonyl compounds to primary amines using NH_3_ and H_2_ over Co-Ph@SiO_2_(900).

Based on reaction facts and some reported knowledge, a plausible mechanism was proposed to address the reductive amination of carbonyl compounds to primary amines using NH_3_ and H_2_ over Co-Ph@SiO_2_(900) (Figure 6) [37]. During the reaction, firstly, imines were rapidly generated from the condensation of carbonyl group and NH_3_ even in the absence of any catalysts. Secondly, the imines were hydrogenated over Co-Ph@SiO_2_(900) to produce primary amines, which could rapidly react with the unreacted carbonyl compounds to form Schiff bases. Finally, in the presence of NH_3_, the generated Schiff bases were further converted into the desired primary amines over Co-Ph@SiO_2_(900).

## 3. Materials and Methods

All chemicals were used as procured without further processing; specifications of the equipment used and the synthesis and testing methods employed are detailed in the Supporting Information.

## 4. Conclusions

In conclusion, we developed a sustainable cobalt-based catalyst (Co-Ph@SiO_2_(900)) for efficient primary amine synthesis via carbonyl reductive amination. Prepared through aqueous-phase immobilization of cobalt–phenanthroline on silica and via 900 °C pyrolysis, this catalyst features uniformly dispersed, graphitic carbon-encapsulated Co nanoparticles with enhanced porosity, acidity, and basicity. These structural attributes enable exceptional performance (>99% conversion, >98% selectivity for acetophenone amination) by synergistically activating key reaction intermediates. The system demonstrates broad substrate versatility (>40 examples), successfully converting sterically hindered ketones, functionalized aldehydes, halogenated aromatics, and biomass-derived molecules while preserving sensitive functional groups (yields up to 99%). Key advantages include a green aqueous synthesis process, facile magnetic separation enabling four-cycle reusability, and broad support compatibility (C, Al_2_O_3_, ZrO_2_, TiO_2_, CeO_2_, Nb_2_O_5_). Mechanistic studies confirm this non-noble metal catalyst overcomes critical selectivity and sustainability challenges in industrial amine production for pharmaceuticals and fine chemicals through a cascade pathway involving rapid carbonyl amination to imine formation, followed by hydrogenation and dynamic Schiff base conversion. We believe that this universal methodology to produce primary amines has great potential for application in the synthesis of functionalized and complex organic molecules for advanced applications in life and material sciences.

## Figures and Tables

**Table 1:** Activity of various catalysts for reductive amination of acetophenone *^a^*.

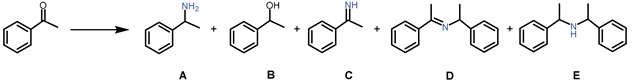							
Entry	Catalyst	Conversion (%)	Selectivity (%)				
			A	B	C	D	E
1	blank	66.0	0	0	>99	0	0
2	precursor	72.7	0	0	>99	0	0
3	Co-Ph@SiO_2_(700)	71.4	36.2	1.1	27.7	35.0	0
4	Co-Ph@SiO_2_(800)	82.6	69.4	1.4	1.8	27.4	0
5	Co-Ph@SiO_2_(900)	92.1	82.2	2.0	1.0	17.5	0
6	Co-Ph@SiO_2_(1000)	65.1	2.2	0	76.0	21.8	0
7 *^b^*	Co-Ph@SiO_2_(900)	>99	98.0	2.0	0	0	0
8	Ni-Ph@SiO_2_(900)	67.1	0	0	95.5	4.5	0
9	Fe-Ph@SiO_2_(900)	68.3	0	0	98.1	1.9	0

*^a^* Reaction conditions: acetophenone, 1 mmol; methanol, 3 mL; H_2_, 3.4 MPa; NH_3_, 0.6 MPa; catalyst, 1.5 mol%; 80 °C; 8 h. *^b^* acetophenone, 1 mmol; methanol, 3 mL; H_2_, 3.4 MPa; NH_3_, 0.6 MPa; catalyst, 3 mol%; 100 °C. Yields were determined by GC with n-butanol as an internal standard. The main reaction conditions for entries 1–9 are identical, except for a slight variation in the catalyst amount for entry 7.

**Table 2:** Reductive amination of various acetophenones over Co-Ph@SiO_2_(900) *^a^*.

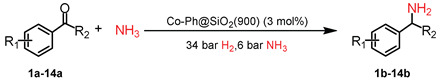			
Entry	Ketone	Product	Yields *^b, e^* (%)
1	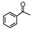 1a	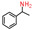 1b	98 (96)
2	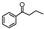 2a	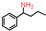 2b	99 (97)
3	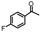 3a	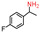 3b	98 (95)
4	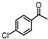 4a	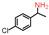 4b	95 (93)
5	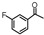 5a	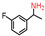 5b	96 (93)
6	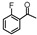 6a	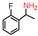 6b	97 (92)
7	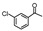 7a	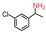 7b	95 (93)
8 *^c^*	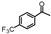 8a	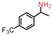 8b	98 (95)
9	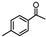 9a	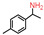 9b	98 (94)
10	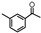 10a	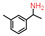 10b	97 (93)
11	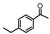 11a	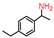 11b	96 (93)
12 *^d^*	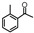 12a	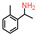 12b	96 (94)
13	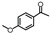 13a	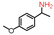 13b	98 (95)
14	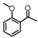 14a	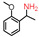 14b	98 (94)

*^a^* Reaction conditions: ketone, 1 mmol; methanol, 3 mL; H_2_, 3.4 MPa; NH_3_, 0.6 MPa; Co-Ph@SiO_2_(900), 40 mg; 100 °C; 8 h. *^b^* The conversion and selectivity were determined by GC using 1-butanol as a standard. *^c^* 24 h. *^d^* 12 h. *^e^* Isolated yield is shown in parentheses.

**Table 3:** Reductive amination of various others ketones over Co-Ph@SiO_2_(900) *^a^.*

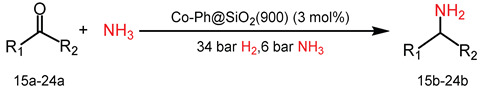			
Entry	Ketone	Product	Yields *^b,d^* (%)
1	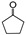 15a	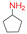 15b	98 (96)
2	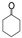 16a	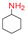 16b	99 (95)
3	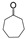 17a	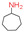 17b	97 (94)
4	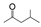 18a	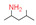 18b	96 (93)
5	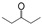 19a	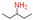 19b	97 (93)
6	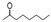 20a	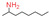 20b	97 (95)
7	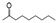 21a	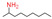 21b	94 (92)
8	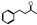 22a	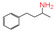 22b	98 (94)
9	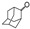 23a	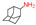 23b	97 (93)
10 *^c^*	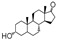 24a	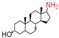 24b	90 (88)

*^a^* Reaction conditions: ketone, 1 mmol; methanol, 3 mL; H_2_, 3.4 MPa; NH_3_, 0.6 MPa; Co-Ph@SiO_2_(900), 40 mg; 100 °C; 8 h. *^b^* The conversion and selectivity were determined by GC using 1-butanol as a standard. *^c^* 24 h. *^d^* Isolated yield is shown in parentheses.

**Table 4:** Reductive amination of various aldehydes over Co-Ph@SiO_2_(900) ^a^.

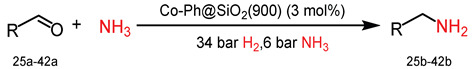			
Entry	Aldehyde	Product	Yields *^b, e^*(%)
1	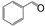 25a	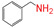 25b	98 (93)
2	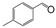 26a	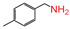 26b	96 (93)
3	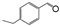 27a	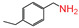 27b	95 (92)
4	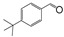 28a	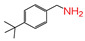 28b	93 (90)
5	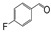 29a	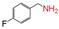 29b	95 (93)
6 *^c^*	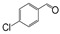 30a	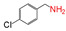 30b	93 (92)
7 *^c^*	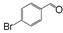 31a	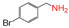 31b	90 (86)
8	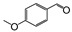 32a	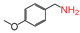 32b	95 (90)
9	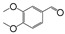 33a	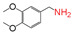 33b	95 (92)
10	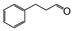 34a	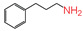 34b	98 (94)
11	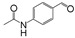 35a	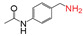 35b	94 (90)
12 *^c^*	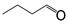 36a	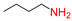 36b	94 (91)
13 *^c^*	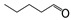 37a	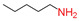 37b	92 (90)
14 *^c^*	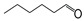 38a	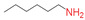 38b	90 (88)
15 *^c^*	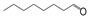 39a	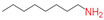 39b	88 (85)
16 *^d^*	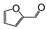 40a	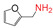 40b	72 (70)
17 *^d^*	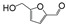 41a	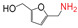 41b	73 (70)
18 *^c^*	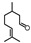 42a	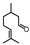 42b	95 (90)

*^a^* Reaction conditions: aldehydes, 1 mmol; methanol, 3 mL; H_2_, 3.4 MPa; NH_3_, 0.6 MPa; Co-Ph@SiO_2_(900), 40 mg; 100 °C; 12 h. *^b^* The conversion and selectivity were determined by GC using 1-butanol as a standard. *^c^* 16 h. *^d^* 24 h. *^e^* Isolated yield is shown in parentheses.

## Data Availability

The data presented in this study are available on request from the corresponding author.

## Supplementary Materials

The following supporting information can be downloaded at https://www.mdpi.com/article/10.3390/molecules30153089/s1: Table S1: Activity of various catalysts for reductive amination of acetophenone. Table S2: ICP–OES analysis of different catalysts and used Co-Ph@SiO_2_ catalysts; Table S3: Summary of the results from N_2_ adsorption-desorption. Table S4: Comparison of Co-Ph@SiO_2_(900) with state-of-the-art catalysts in reductive amination of aldehydes and ketones into primary amines; Figure S1: SEM images of the prepared Co-Ph@SiO_2_(900). Figure S2: TEM images and corresponding size distribution of (A) Co-Ph@SiO_2_(700), (B) Co-Ph@SiO_2_(800), (C) Co-Ph@SiO_2_(900) and (D) Co-Ph@SiO_2_(1000); (eighty particles were counted to obtain the histograms). Figure S3: TEM images of Co-Ph@SiO_2_(1000). Figure S4: TEM and EDS mapping images of the prepared Co-Ph@SiO_2_(900). Figure S5: Survey spectra of different Co-Ph@SiO_2_(x). Figure S6: NH_3_-TPD profile (A) and acidic sites quantity (B) of Co-Ph@SiO_2_(900) and Co@SiO_2_(900). Figure S7: The activation of C=N groups in in situ generated imines and Schiff bases by the acidic sites on Co-Ph@SiO_2_(900). Figure S8: CO_2_-TPD profile (A) and basic sites quantity (B) of Co-Ph@SiO_2_(900) and Co@SiO_2_(900). Figure S9: The hydrogenation steps in the reaction process. Figure S10: Magnetic separation of the catalyst after reaction. Figure S11: SEM image, TEM image, XPS spectra of Co 2p and N 1s for the recovered Co-Ph@SiO_2_(900). Figure S12: Powder XRD patterns for (a) fresh Co-Ph@SiO_2_(900), (b) recycled Co-Ph@SiO_2_(900). Figure S13: Control experiments using Schiff base as the reactant over Co-Ph@SiO2(900). Reaction conditions: methanol, 3 mL; Co-Ph@SiO_2_(900), 40mg; 100 °C; 8 h.; Figure S14: GC-MS spectra of imidazoline derived from the reductive amination of furfural catalyzed by Co-Ph@SiO_2_(900).
