# Supplementary material for: Simple Cobalt Nanoparticle-Catalyzed Reductive Amination for Selective Synthesis of a Broad Range of Primary Amines
Source: Molecules. 2025 Jul 23;30(15):3089. doi: 10.3390/molecules30153089 (PMC12348235; doi:10.3390/molecules30153089)

Supporting Information for:

Simple Cobalt Nanoparticles-Catalyzed Reductive Amination for Selective Synthesis of a Broad Range of Primary Amines

Bingxiao Zheng *, Liqin Yang, Yashuang Hei, Ling Yu, Sisi Wen, Lisi Ba, Long Ao, and Zhiju Zhao *

Functional Polymer Materials R&D and Engineering Application Technology Innovation Center of Hebei Province, Xingtai University, Xingtai, Hebei 054001, China; 15116903609@163.com (B.Z.); yanglq2005@163.com (L.Y.); heiys0815@163.com (Y.H.); 200820347@xttc.edu.cn (L.Y.); 18844115900@163.com (S.W.) balisi814@163.com (L.B.); 18131425341@163.com (L.A.); 201610339@xttc.edu.cn (Z.Z.)

* Correspondence: 15116903609@163.com(B.Z.); 201610339@xttc.edu.cn (Z.Z.) ; Tel.:+86-151-1690-3609(B.Z.)

Table of Contents

1. Experimental Section. ……………………………………………..…S2
2. Supporting results…………………………………………………S5
3. Chromatograms of GC-measurements.…………..…………..……….....S14

Experimental Section

Materials.

Cyclohexanone (99.8%), and 1-butanol (99.5%) were achieved from Acros. 3-Pentanone (98%), 4-methyl-2-pentanone (99%), cyclopentanone (99%), 2-nonanone (99%), 4-ethylbenzaldehyde (98%), acetophenone (99%), 4-tert-butylbenzaldehyde (97%), benzaldehyde (99%), 4-ethylacetophenone (>97%), 3-methylacetophenone (98%), 3-chloroacetophenone (>98%), 2-methylacetophenone (98%), 2-fluoroacetophenone (98%), 3-fluoroacetophenone (98%), 4-chloroacetophenone (97%), 4-fluoroacetophenone (98%), 4-(trifluoromethyl)acetophenone (98%), 1,10-phenanthroline (99%) 2-methoxyacetophenone (≥98%), 4-methylacetophenone (98%) and butyrophenone (99%) were purchased from Aladdin. Butyraldehyde (99%), cycloheptanone (99%), hexanal (99%), 2-octanone (99%), n-octanal (99%) and *p*-anisaldehyde (99%) were achieved from TCI. 3-Phenylpropanal (99%) was purchased from ARK. 4-Phenyl-2-butanone (98%), *p*-methyl benzaldehyde (98%), 3-pyridinecarboxaldehyde (98%), 4-fluorobenzaldehyde (98%), 4-chlorobenzaldehyde (98%), *p*-bromo benzaldehyde (99%), veratraldehyde (98%), (±)-citronellal (96%), methyl *p*-formylbenzoate (98%), 4-acetamidobenzaldehyde (98%), furfural (99%), 5-hydroxymethyl-2-furaldehyde (98%), 4-methoxyacetophenone (98%), androsterone (98%) and 2-adamantanone (98%) were achieved from Innochem. Methanol (99.5%) was obtained from Sinopharm. Cobalt(II) acetate tetrahydrate was obtained from Alfa. Silicon dioxide (LUDOX(R) AS-40 colloidal silica 40 wt. % suspension in H_2O) was got from Aldrich.

Instruments. Conversions and yields were determined by a gas chromatography (Agilent 8890B) with the FID detector and a HP-5 column (30 m x 320 μm x 0.25μm).

Catalyst Characterization. Inductively coupled plasma atomic emission spectrometry (ICP-AES) was adopted to measure the actual Co content. X-ray diffraction (XRD) patterns were obtained using Rigaku and model with Cu Kα radiation (1.5418 Å). X-ray photoelectron spectroscopy (XPS) study was determined on the AXIS Supra surface analysis instrument with an X-ray monochromatic source (combined Al/Ag anode, energy 1486.6/2984.2eV) and studies were performed in 10-9 mbar vacuum. The morphology of the electrodes was recorded on

Hitachi S4800 scanning electron microscope (SEM) at 3 kV and transmission electron microscopy (TEM) (Tecnai G2 F30 FETEM). Nitrogen adsorption−desorption analysis at 77 K was measured on a Quantachrome Autosorb-3B system after the catalyst was outgassed under vacuum at 100 °C for 12 h. The specific surface area was determined according to the Brunauer–Emmett–Teller (BET) equation from the adsorption branch.

Synthesis of Co-Ph@SiO$_2$(x). Solution A containing Co(OAc)$_2$•4H$_2$O (1.5 mmol) and 1,10-phenanthroline (3 mmol) in 10 mL distilled water under stirring condition at room temperature. Solution B contains LUDOX® AS-40 colloidal silica (2.5 g) in 40 mL distilled water. Then, solution A were dropwise added to solution B, and the whole reaction mixture was heated at 60 °C for 4 h and then water was removed under evaporation by heating at 60 °C. The obtained Co(phen)$_2$(OAc)$_2$@SiO$_2$ was grinded into powder and pyrolyzed at 900 °C (or 700, 800, 1000 °C) for 2 h under N_2 atmosphere. After cooling to room temperature, the materials were donated as Co-Ph@SiO$_2$**(x)** (x = pyrolysis temperature).

General procedures to conduct the reaction. In a typical route, the desired carbonyl compound and catalyst were charged into a stainless steel reactor with a Teflon coating (14 mL inner volume). After the reactor was sealed, it was purged with 0.5 MPa NH_3 three times to remove residual air. After that, the NH_3 was charged into the reactor to reach a desired pressure. Then, the reactor was further pressurized with H_2. Then, the autoclave was placed into a constant-temperature air bath and heated to the desired temperature, and the reactions were conducted with a stirring rate of 800 rpm for the desired reaction time. After the reaction, the products were analyzed quantitatively using n-butanol as the internal standard (cyclohexanol for the case with butyraldehyde as the substrate) by gas chromatography (GC) and gas chromatography coupled with mass spectroscopy (GC-MS). And the obtained solution was then separated using the flash chromatography system with an amino column (eluent, MeOH and CH_3Cl or AcOEt). The filtrate was evaporated to obtain the analytically pure products.

Recycling of catalyst. The recyclability of the Co-Ph@SiO_2(900) catalyst was tested for reductive amination maintaining the same reaction conditions as described above, except using the recovered catalyst. Each time, the catalyst was isolated from the reaction mixture by magnetic separation at the end of catalytic reaction, thoroughly washed with methanol, and then dried in vacuum oven at 60 °C overnight.

Synthesis of Co/SiO_2. Solution A containing $Co(OAc)_2$•$4H_2O$ (1.5 mmol) in 10 mL distilled water under stirring condition at room temperature. Solution B contains LUDOX® AS-40 colloidal silica (2.5 g) in 40 mL distilled water. The mixture was heated at 60 °C for 4 h and then water was removed under evaporation by heating at 60 °C. The obtained product was then transferred to a furnace and pyrolyzed for 3 h at 800 °C for 3 h at the heating rate of 5 °C/min under flowing nitrogen and then naturally cooled to room temperature to obtain the Co/SiO_2.

Table S1. Activity of various catalysts for reductive amination of acetophenone[a].

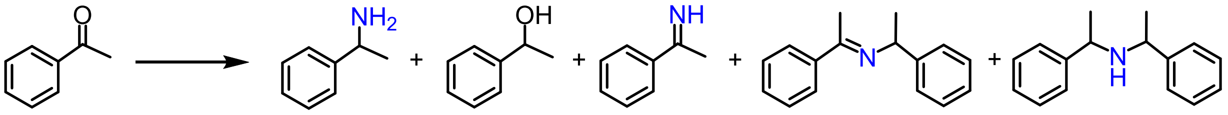

A B C D E

entry	catalyst	conversion(%)	Selectivity (%)				
			A	B	C	D	E
**1**	**Co-Ph@C(900)**	100	95.0	5	0	0	0
**2**	**Co-Ph@Nb_2O_5(900)**	100	96.0	4	0	0	0
**3**	**Co-Ph@TiO_2(900)**	100	97.0	3	0	0	0
**4**	**Co-Ph@Al_2O_3(900)**	100	98.0	2	0	0	0
**5**	**Co-Ph@Zr_2O_5 (900)**	100	93.0	7	0	0	0
**6**	**Co-Ph@CeO_2(900)**	100	95.0	5	0	0	0

[a] Reaction conditions: acetophenone, 1 mmol; methanol, 3 mL; H_2, 3.4 MPa; NH_3, 0.6 MPa; catalyst, 1.5 mol%; 100 °C; 8 h.

Table S2. ICP–OES analysis of different catalysts and used Co-Ph@SiO_2 catalysts

entry	Catalyst	Co content (wt%)
**1**	Co-Ph@SiO_2(700)	4.16
**2**	Co-Ph@SiO_2(800)	4.35
**3**	Co-Ph@SiO_2(900)	4.69
**4**	Co-Ph@SiO_2(1000)	5.45
**5**	Co-Ph@ SiO_2(900) (1 cycle)	4.60
**6**	Reaction solution	0
**7**	Co@SiO_2(900)	7.18
**8**	Co@SiO_2(900)	6.88
**9**	Co-Ph@C(900)	5.16
**10**	Co-Ph(900)	14.9
**11**	Co-Ph@CeO_2(900)	5.31
**12**	Co-Ph@Al_2O_3(900)	4.78
**13**	Co-Ph@TiO_2 (900)	5.51
**14**	Co-Ph@Nb_2O_5 (900)	5.73
**15**	Co-Ph@ZrO_2(900)	3.84

Table S3. Summary of the results from N_2 adsorption-desorption

entry	Catalyst	S_{BET} (m^2 g^{-1})	Pore volume (cc/g)	Pore Radius Dv(r) (nm)
1	Co-Ph@SiO_2(700)	142.489	0.049	15.563
2	Co-Ph@SiO_2(800)	148.573	0.032	16.372
3	Co-Ph@SiO_2(900)	128.125	0.062	14.832
4	Co-Ph@SiO_2(1000)	86.462	0.069	17.271
5	Co@SiO_2(900)	62.624	0.374	92.768

Table S4. Comparison of Co-Ph@SiO_2(900) with state-of-the-art catalysts in reductive amination of aldehydes and ketones into primary amines.

Catalyst	T (°C)	Time (h)	mol% metal based on substrate	TOF (h^{-1})	Product	Yield(%)	Ref.
Co-DABCO-TPA@C-800	120	15	3.5	1.7	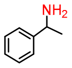	92	24
Co-NPs	50	20	1.5	3.3	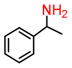	99	25
Ni-TA@SiO_2-800	120	24	1.5	2.5	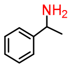	92	26
Ni/Al_2O_3	80	20	2.8	1.7	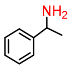	99	27
Fe/(N)SiC	140	20	2.8	0.5	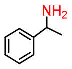	99	28
Ni_1Al_2-$Cs_{1.0}$	100	3.5min	21	78.1	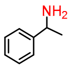	76	30
Fe–C/MgO	100	20	12	0.34	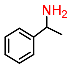	82	31
Co_2P NRs	110	20	10	0.36	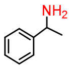	73	32

Fe_2P NC	150	3	10	3.13	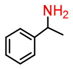	94	33
Co-Ph@SiO_2(900)	100	8	1.5	8.3	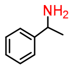	98	This work

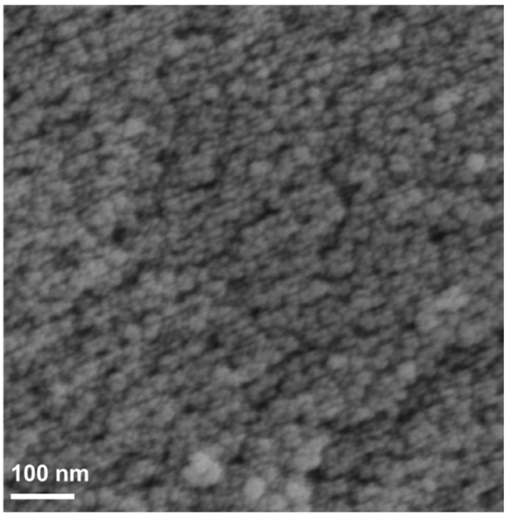

Figure S1. SEM images of the prepared Co-Ph@SiO_2(900).

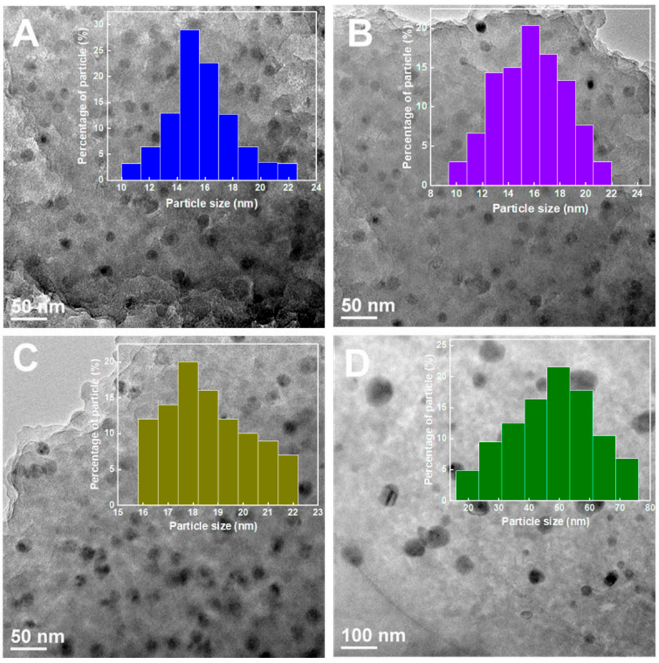

Figure S2. TEM images and corresponding size distribution of (A) Co-Ph@SiO_2(700), (B) Co-Ph@SiO_2(800), (C) Co-Ph@SiO_2(900) and (D) Co-Ph@SiO_2(1000); (eighty particles were counted to obtain the histograms).

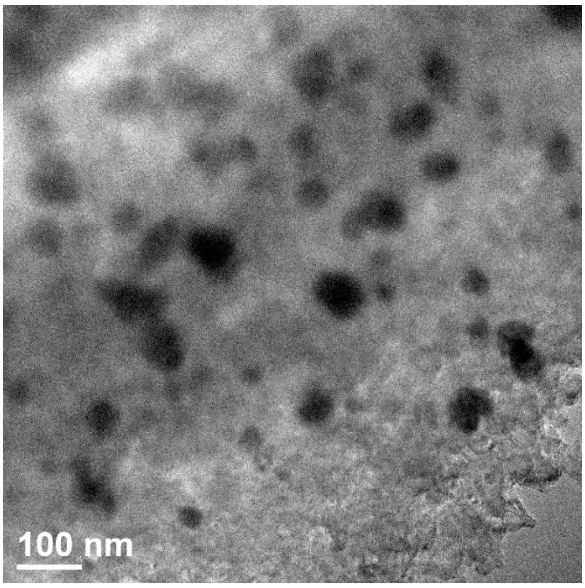

Figure S3. TEM images of Co-Ph@SiO_2(1000)

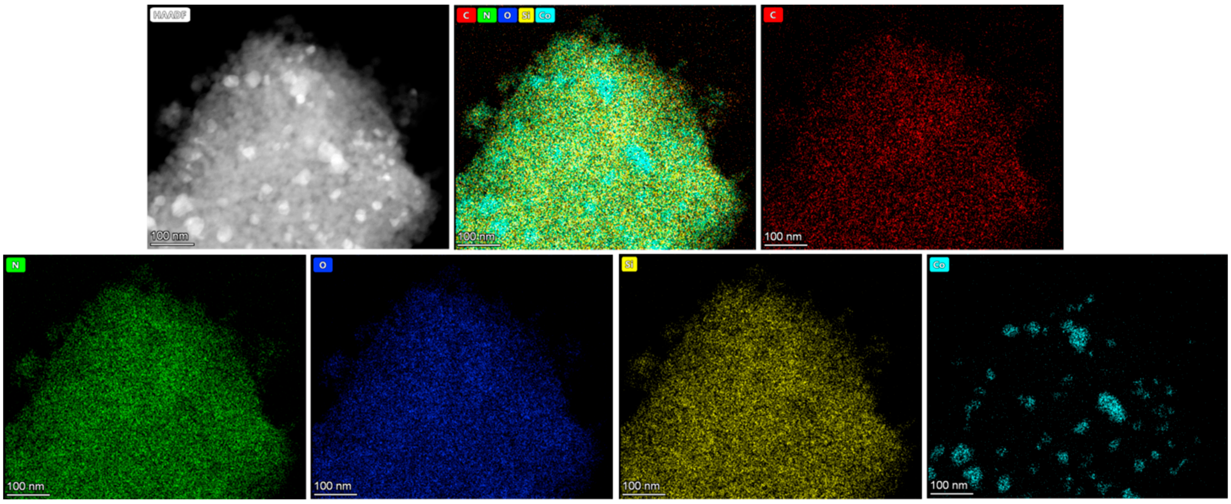

Figure S4. TEM and EDS mapping images of the prepared Co-Ph@SiO_2(900).

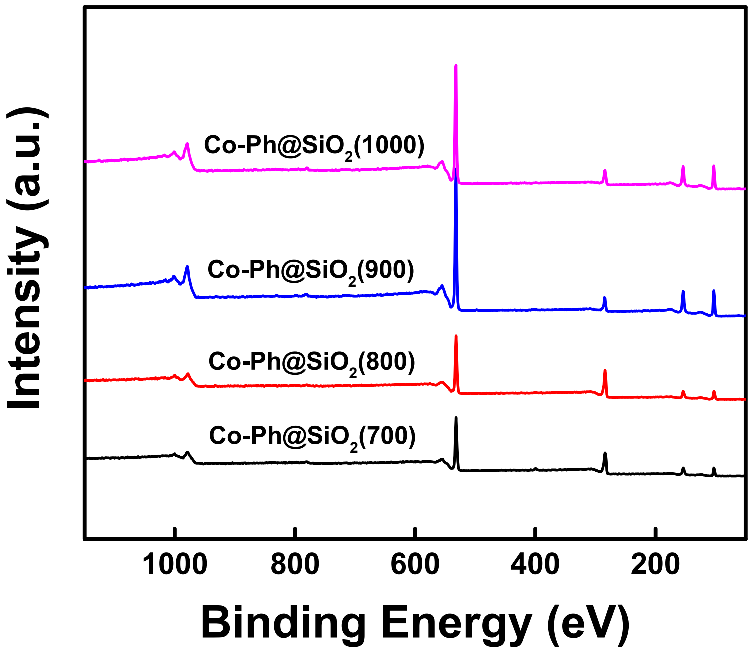

Figure S5. Survey spectra of different Co-Ph@SiO_2(x)

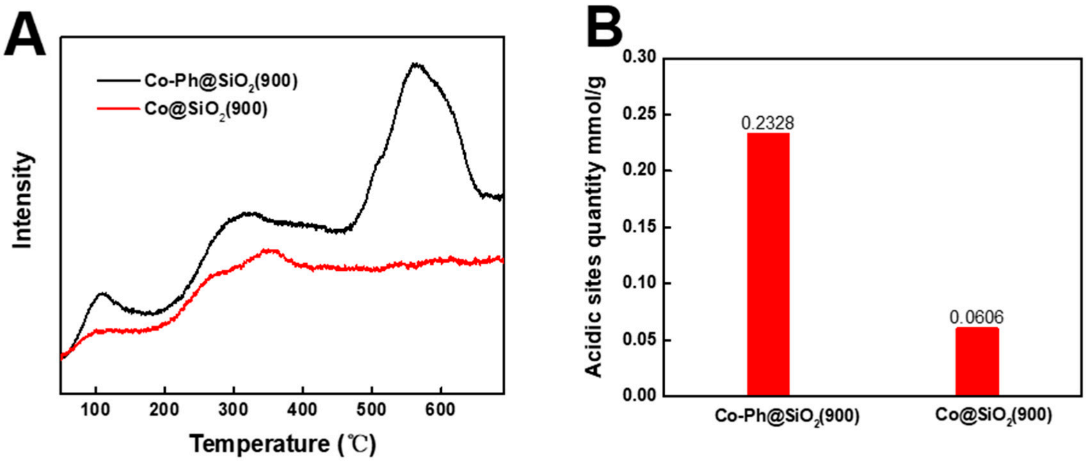

Figure S6. NH_3-TPD profile (A) and acidic sites quantity (B) of Co-Ph@SiO_2(900) and Co@SiO_2(900).

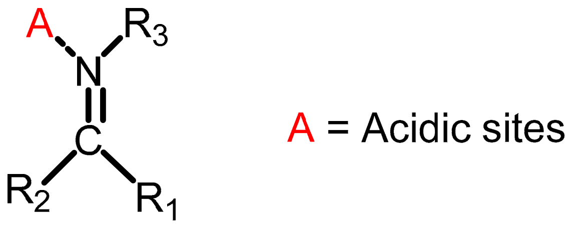

Figure S7. The activation of C=N groups in in situ generated imines and Schiff bases by the acidic sites on Co-Ph@SiO_2(900)

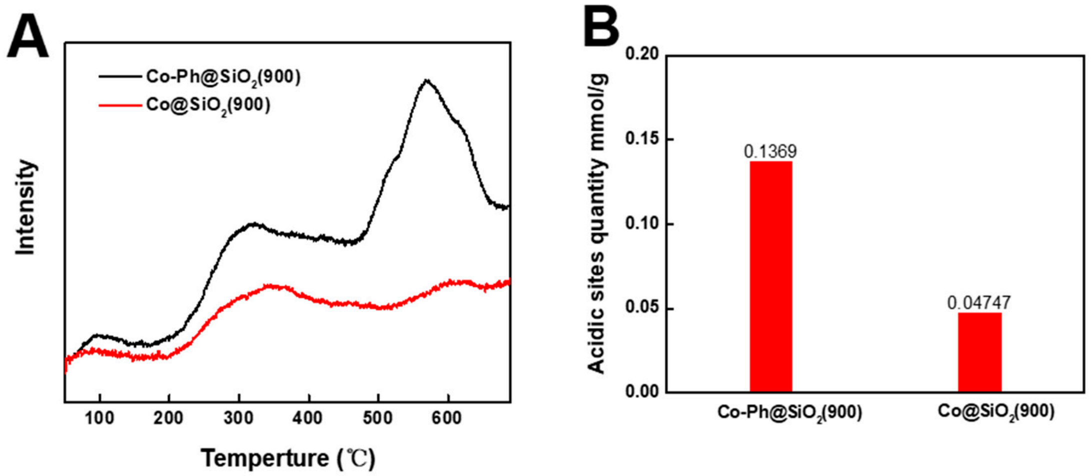

Figure S8. CO_2-TPD profile (A) and basic sites quantity (B) of Co-Ph@SiO_2(900) and Co@SiO_2(900).

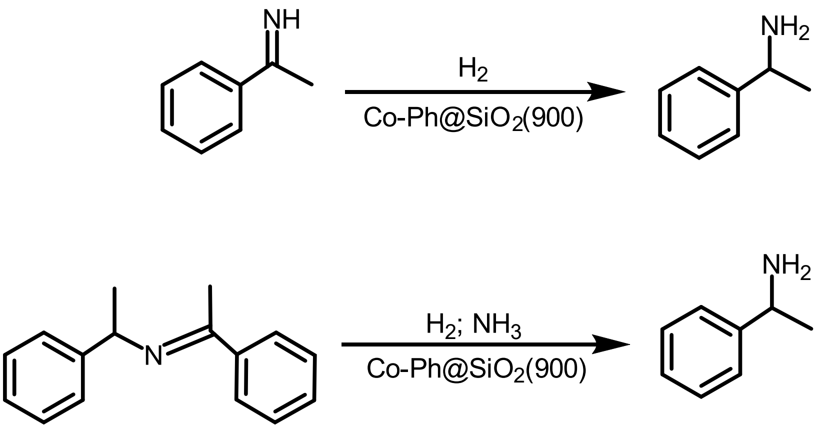

Figure S9. The hydrogenation steps in the reaction process.

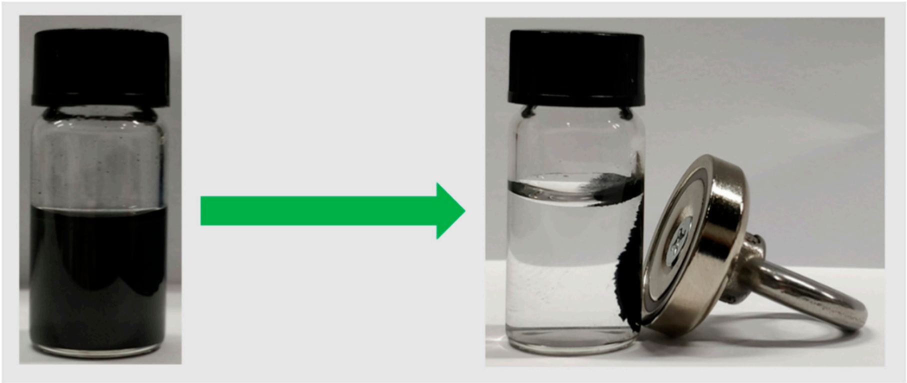

Figure S10. Magnetic separation of the catalyst after reaction

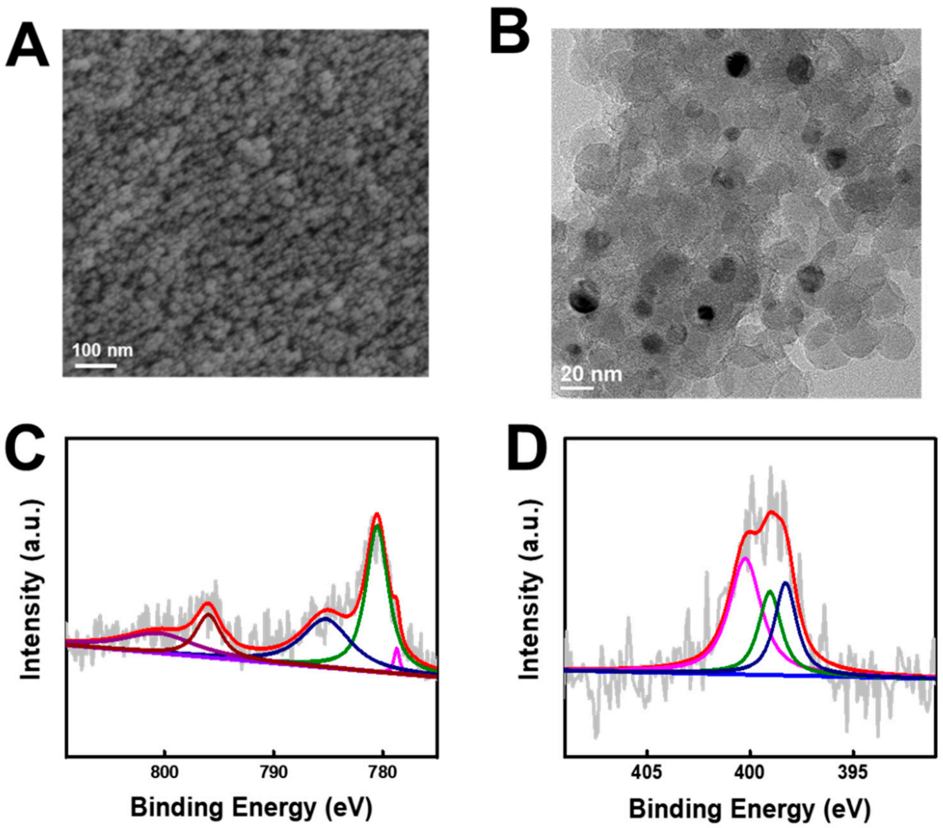

Figure S11. SEM image, TEM image, XPS spectra of Co 2p and N 1s for the recovered Co-Ph@SiO_2(900).

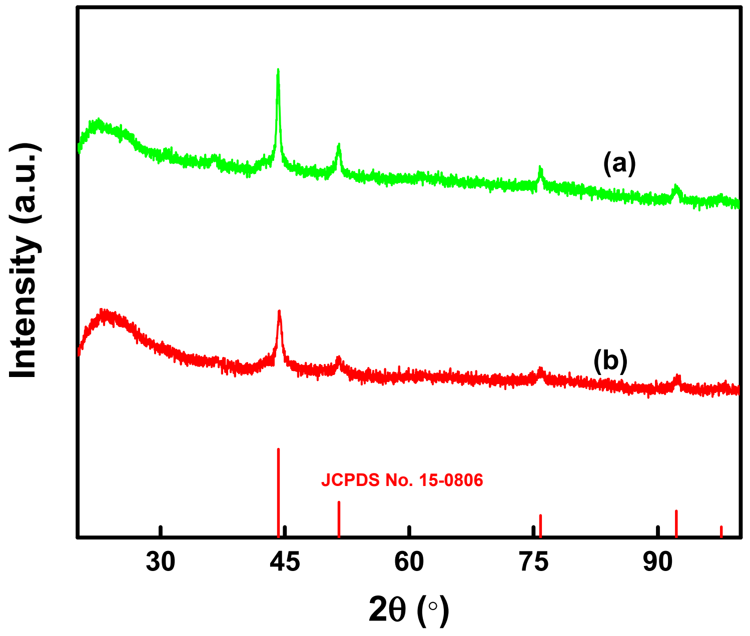

Figure S12. Powder XRD patterns for (a) fresh Co-Ph@SiO_2(900), (b) recycled Co-Ph@SiO_2(900

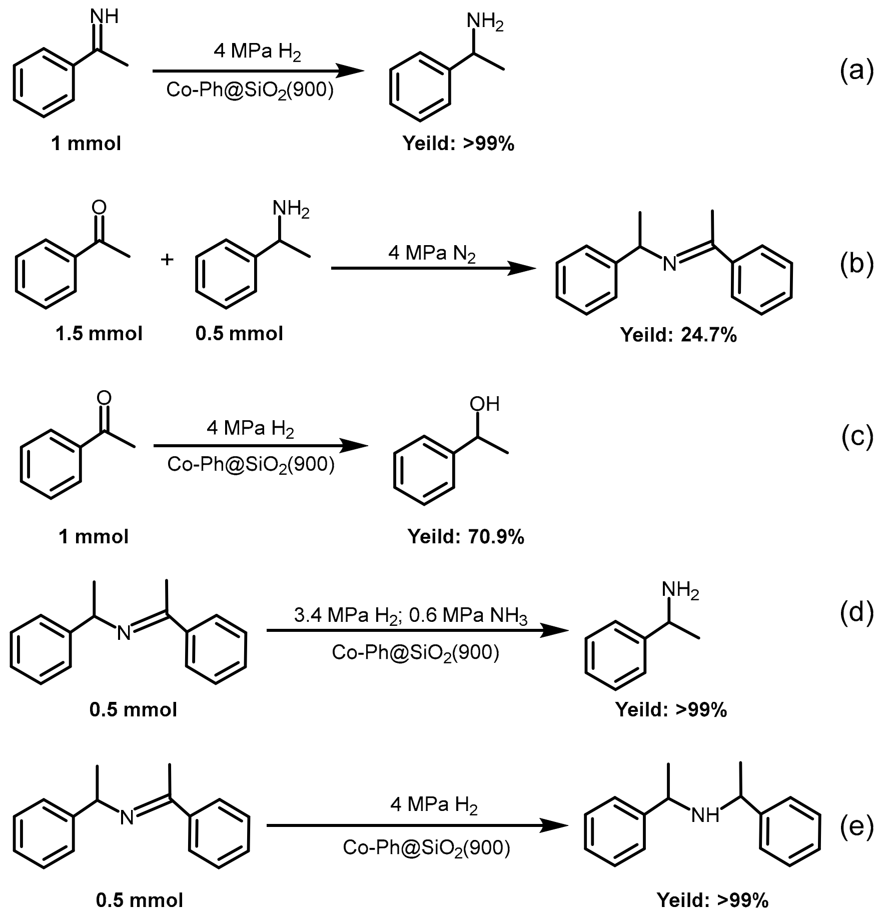

Figure S13. Control experiments using Schiff base as the reactant over Co-Ph@SiO_2(900). Reaction conditions: methanol, 3 mL; Co-Ph@SiO_2(900), 40mg; 100℃; 8 h.

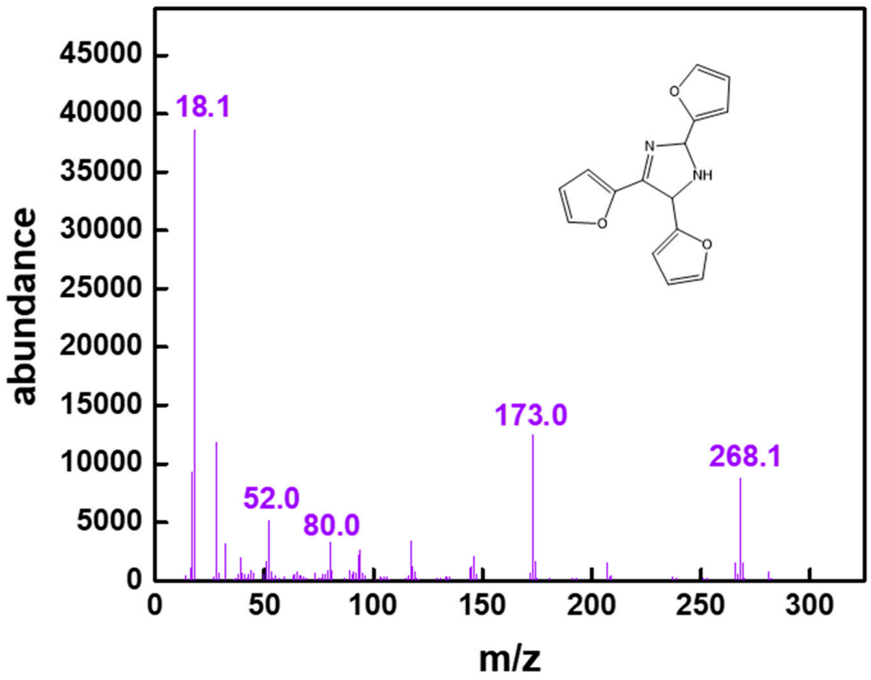

Figure S14. GC-MS spectra of imidazoline derived from the reductive amination of furfural catalyzed by Co-Ph@SiO_2(900).

Chromatograms for GC-measurements

Phenylethan-1-amine

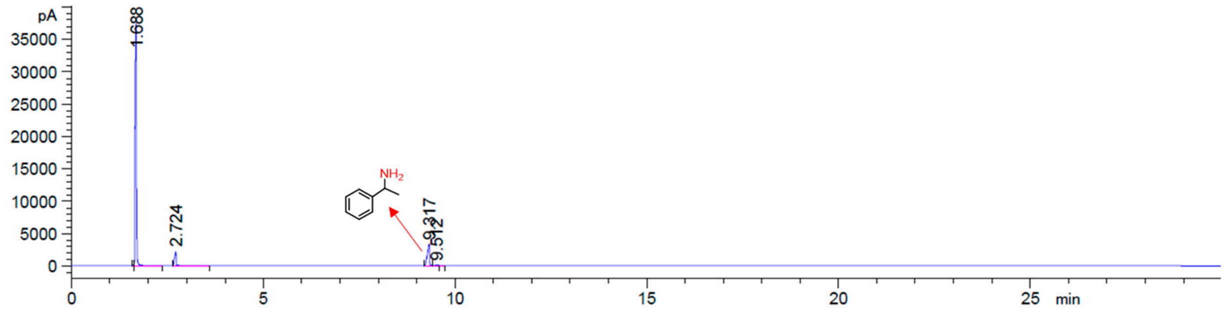

1-(4-Fluorophenyl)ethanamine

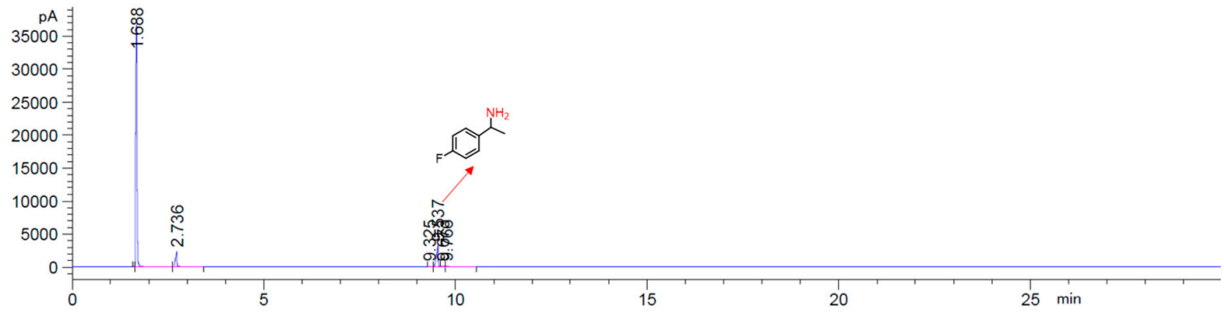

1-(3-Fluorophenyl)ethanamine

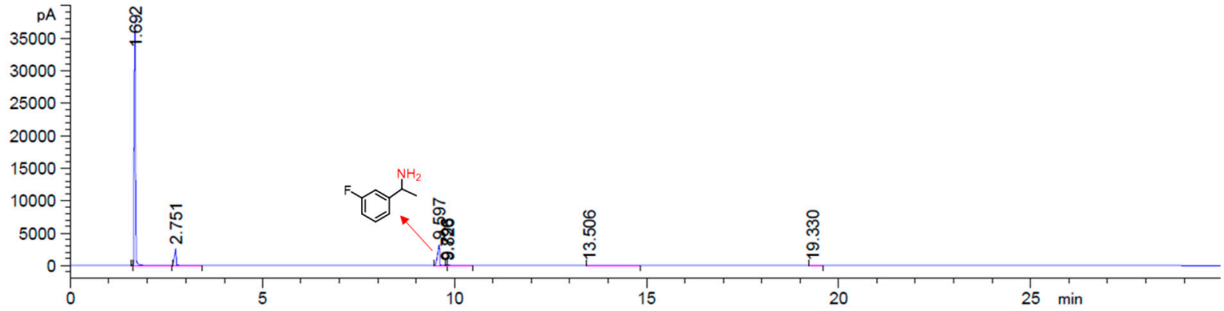

1-(2-Fluorophenyl)ethanamine

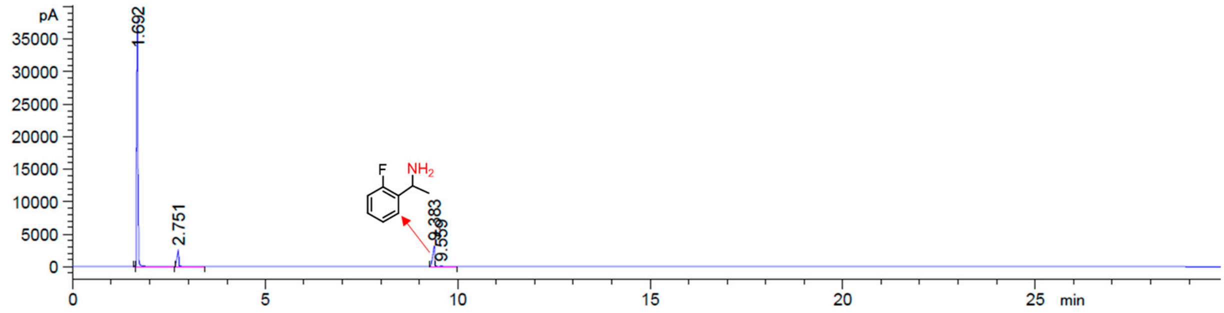

1-(4-Chlorophenyl)ethylamine

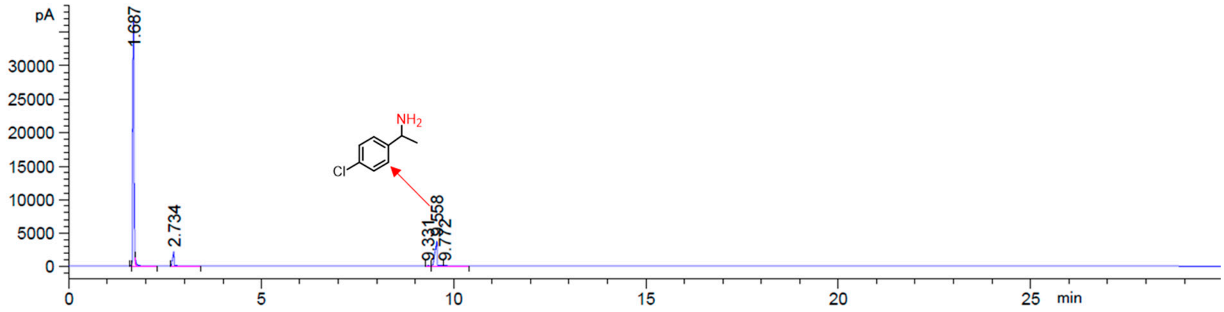

1-(3-Chlorophenyl)ethylamine

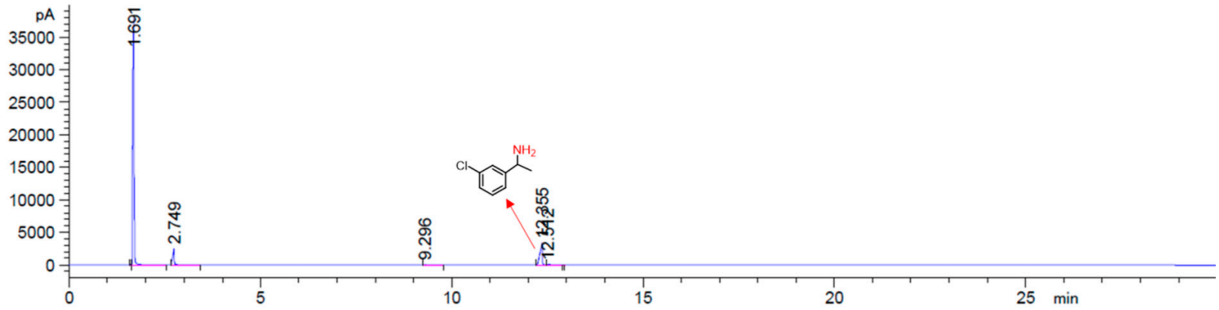

1-(4-Bromophenyl)ethylamine

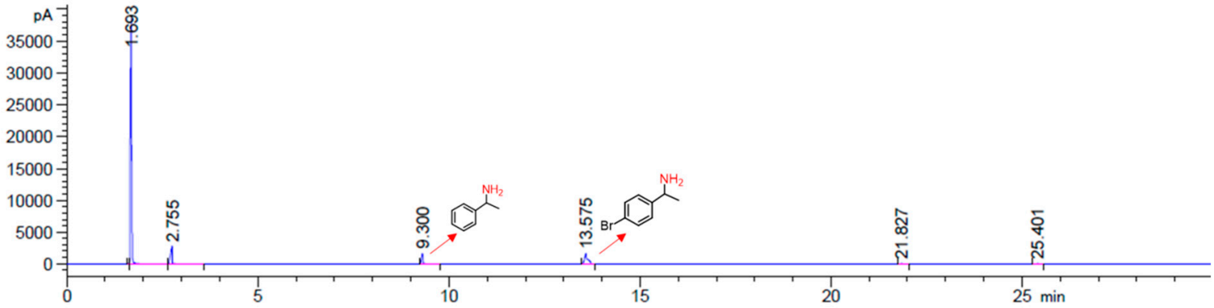

1-(4-(Trifluoromethyl)phenyl)ethanamine

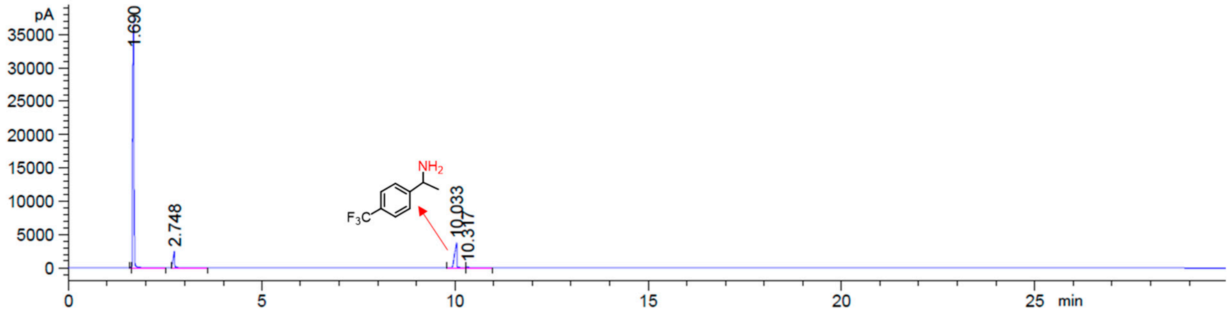

1-(4-Methylphenyl)ethylamine

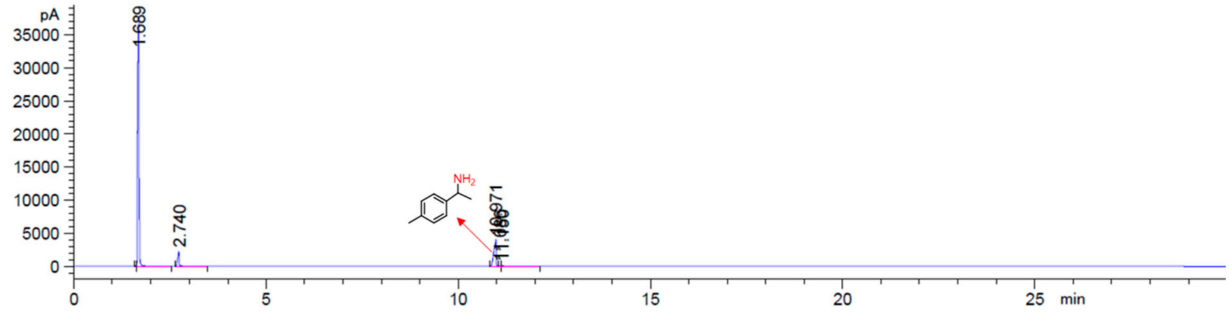

1-(3-Methylphenyl)ethylamine

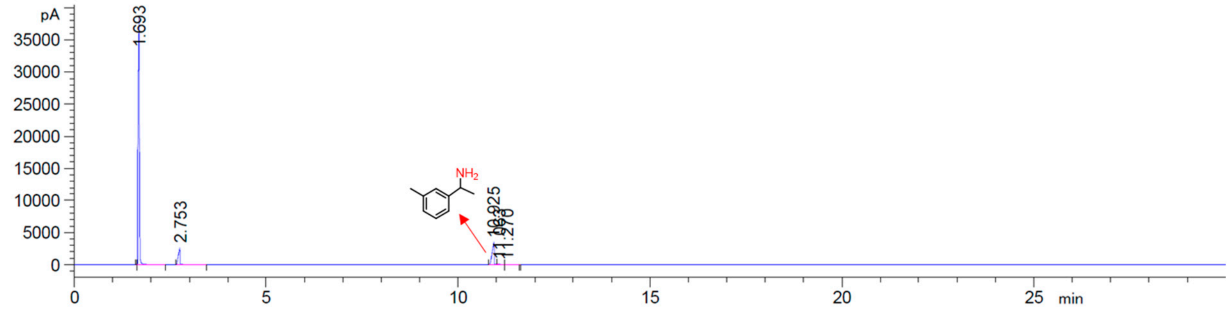

1-(2-Methylphenyl)ethylamine

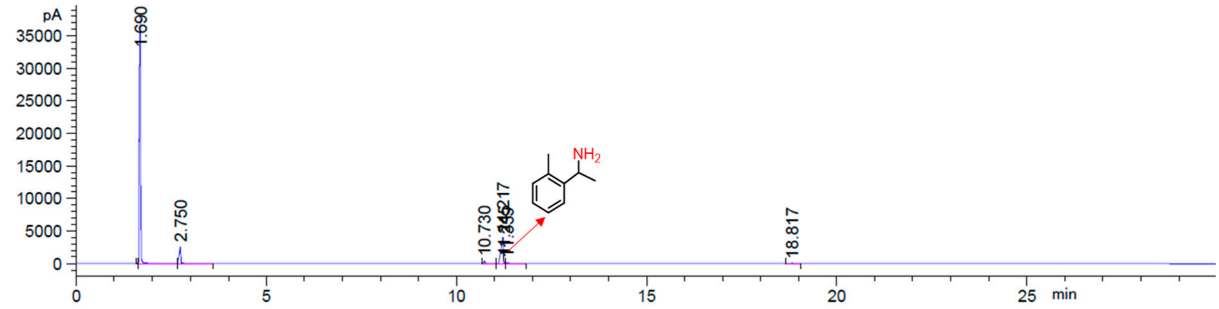

1-(4-Ethylphenyl)ethanamine

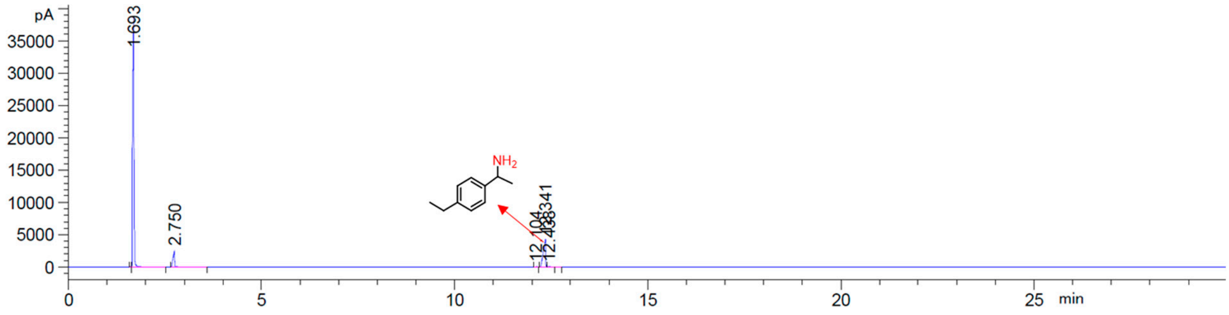

1-(4-Methoxyphenyl)ethylamine

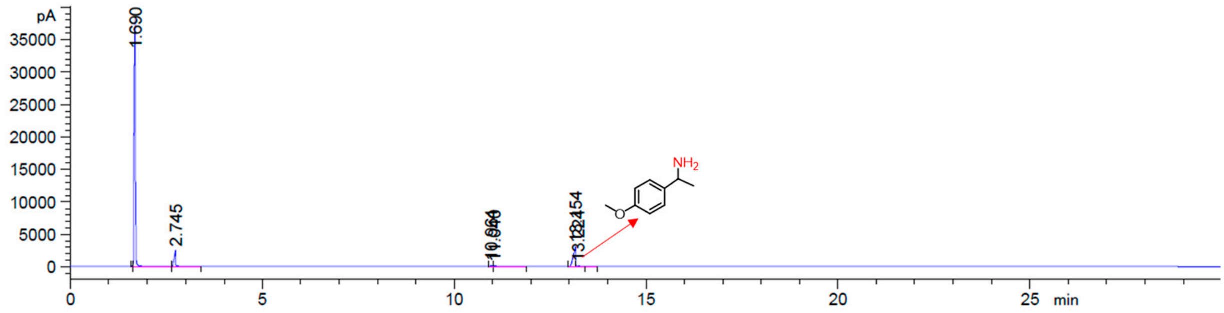

1-(2-Methoxyphenyl)ethylamine

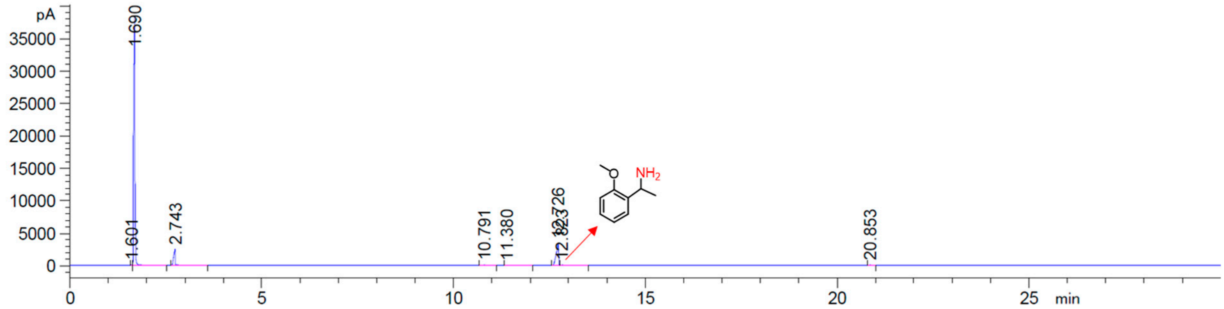

1-phenylbutan-1-amine

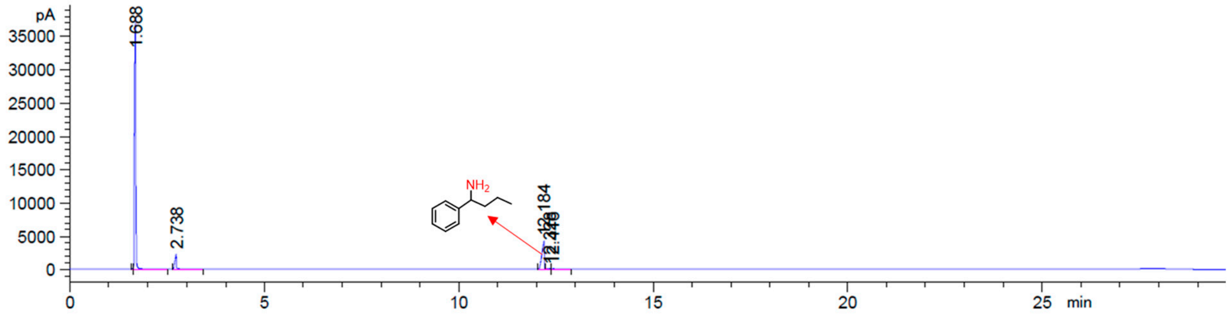

Cyclopentylamine

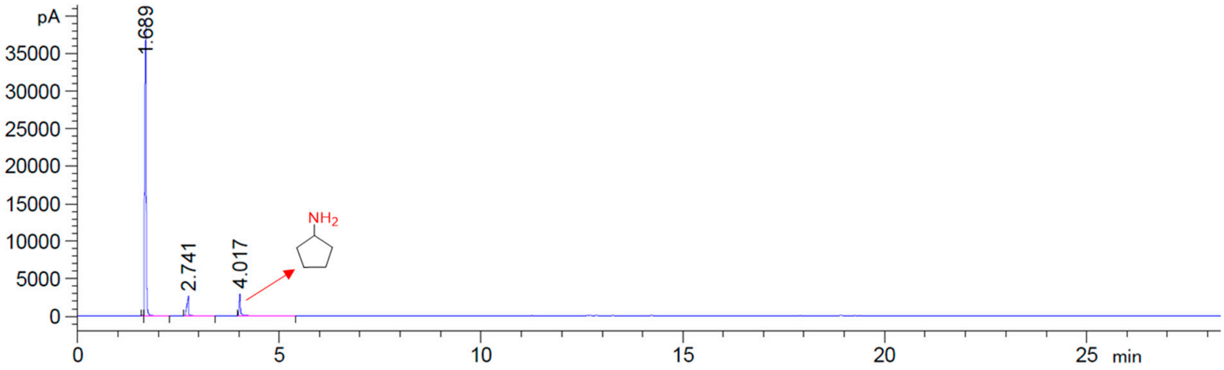

Cyclohexanamine

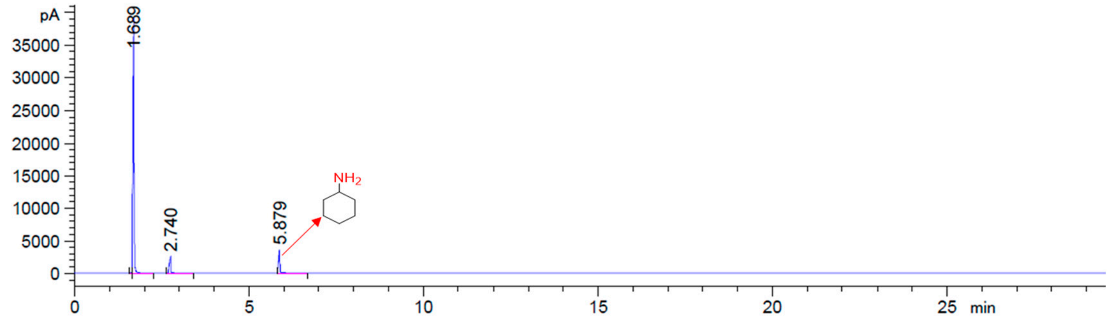

Cycloheptanamine

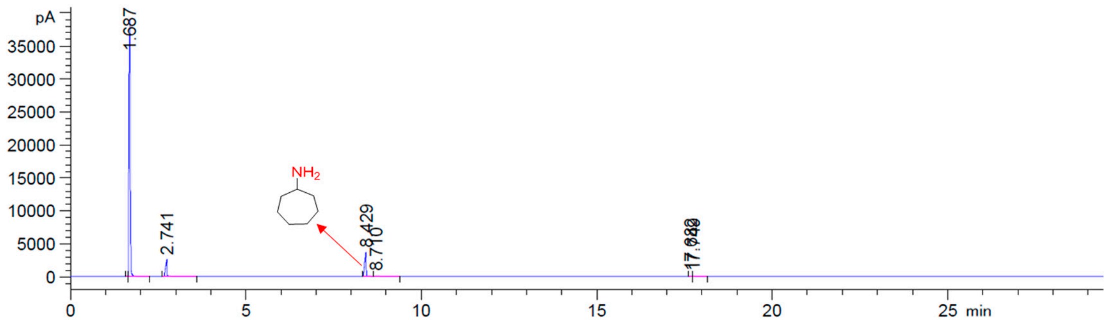

4-Methylpentan-2-amine

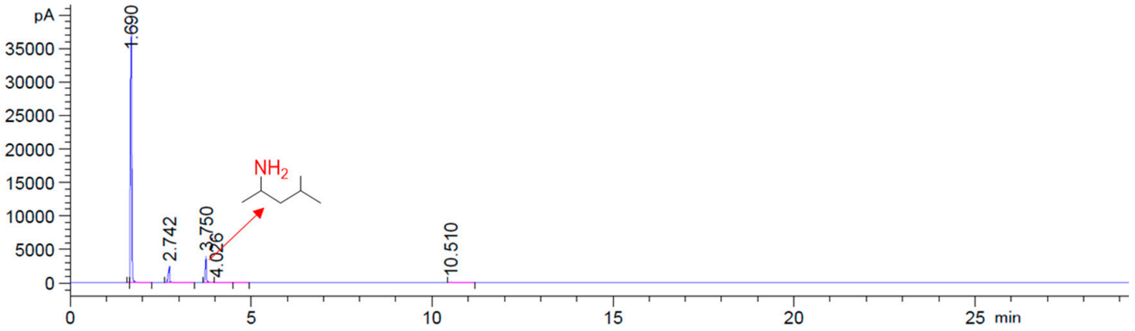

Pentan-3-amine

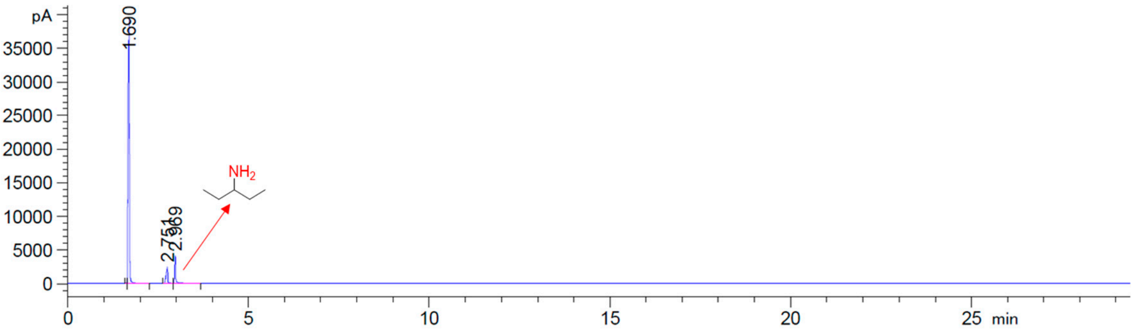

Octan-2-amine

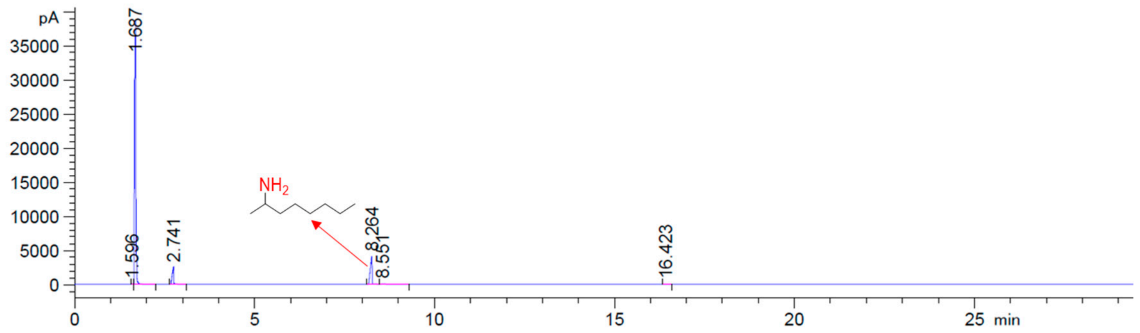

Nonan-2-amine

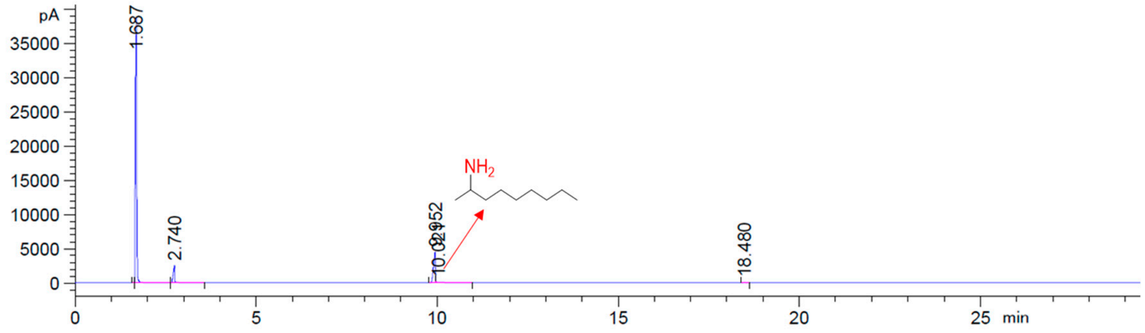

4-Phenylbutan-2-amine

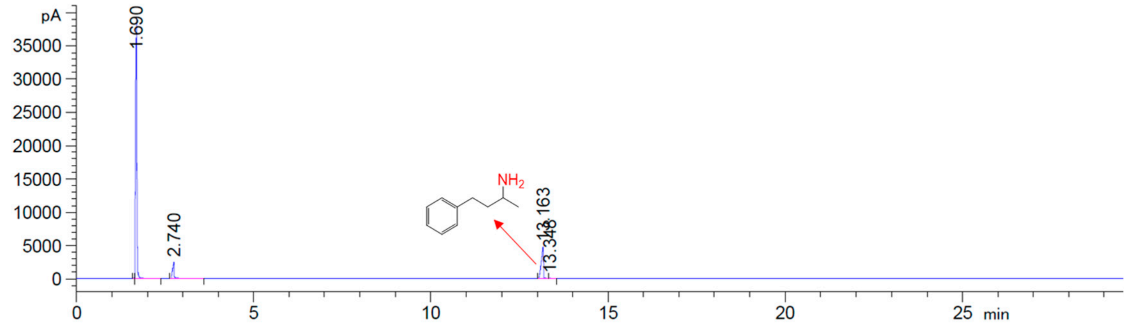

1-Adamantanamine

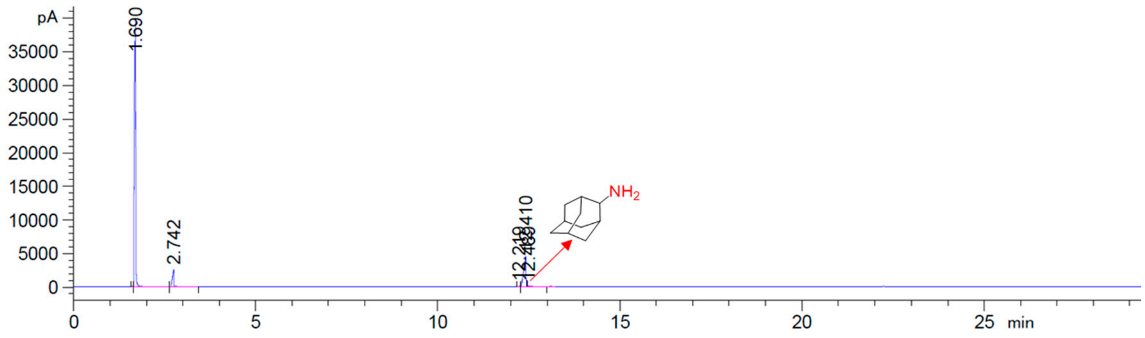

Androsterone-NH_2

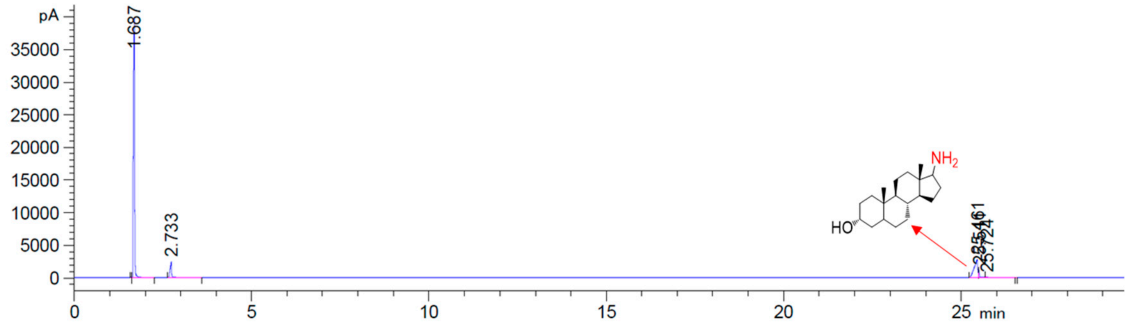

Phenylmethanamine

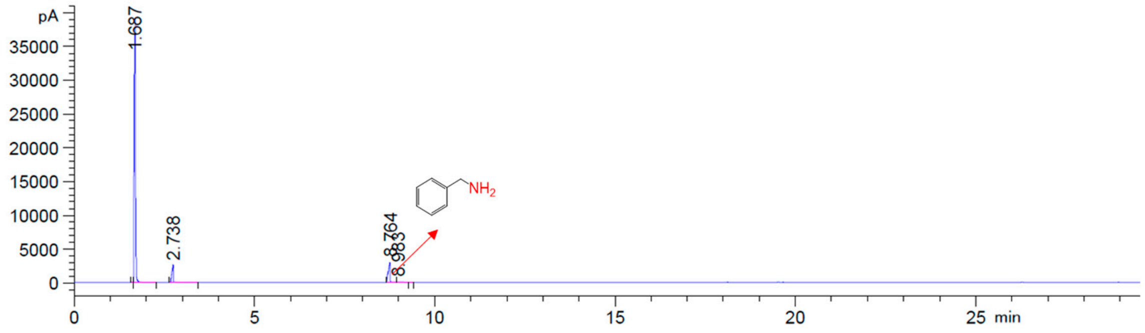

4-Methylbenzylamine

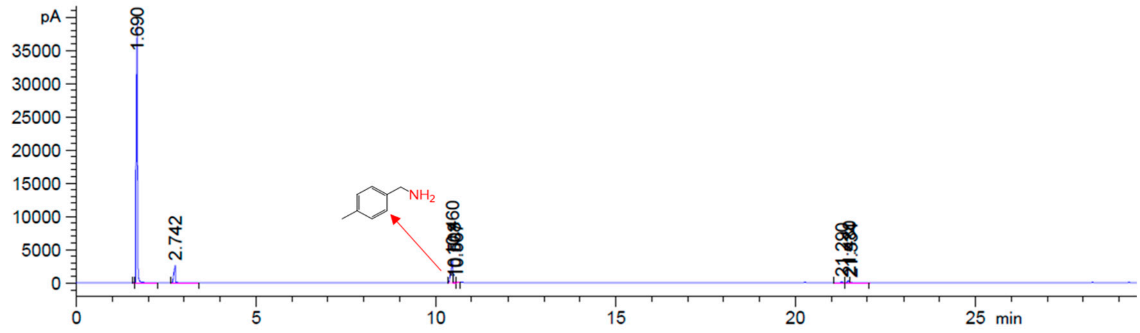

4-Ethylbenzylamine

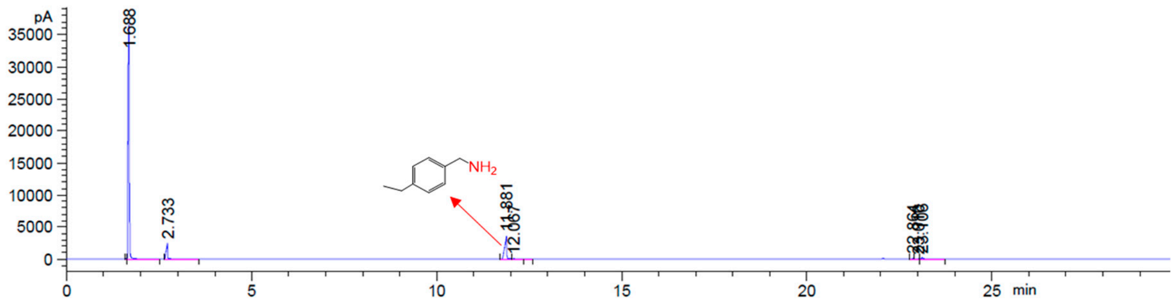

4-tert-Butylbenzylamine

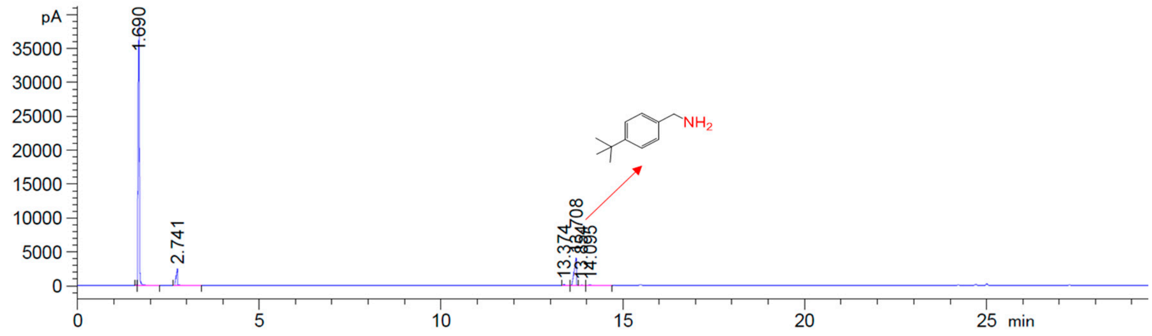

4-Fluorobenzylamine

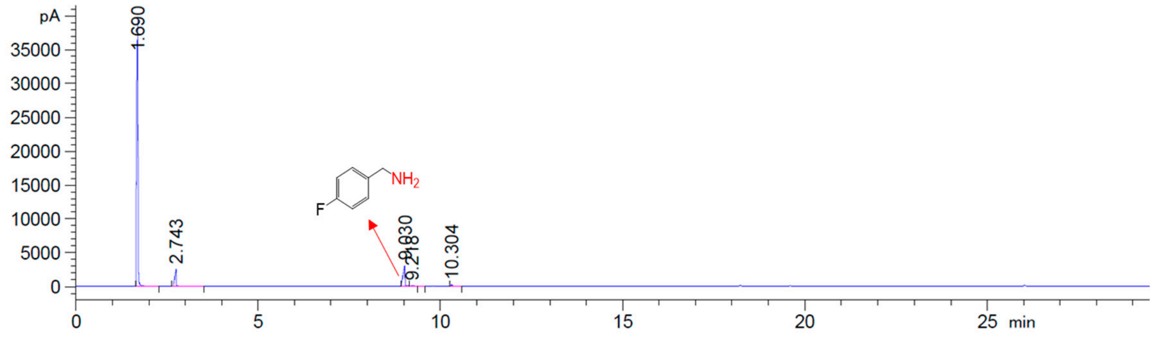

4-Chlorobenzylamine

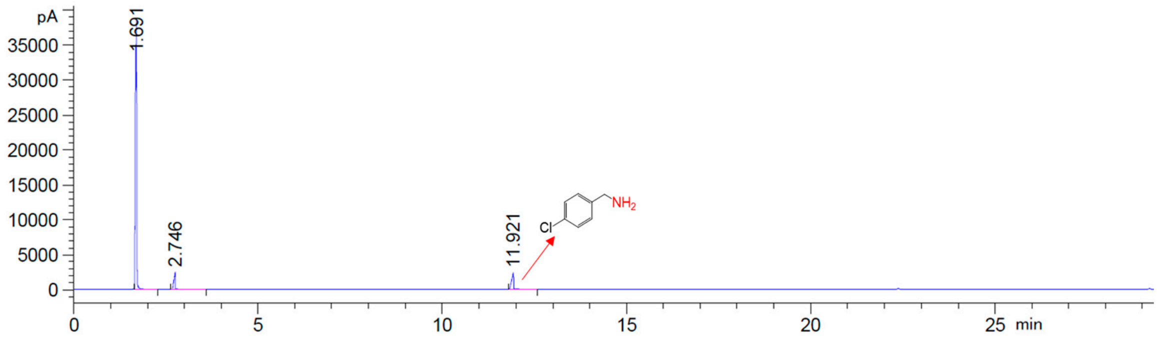

4-Bromobenzylamine

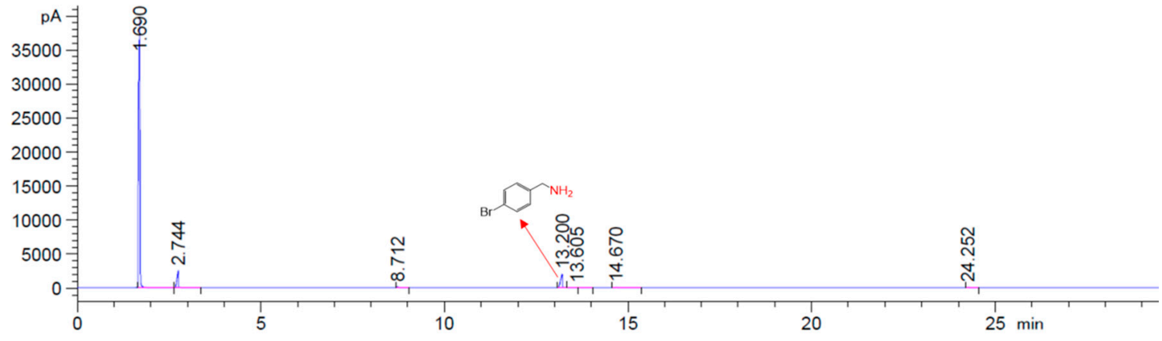

4-Methoxybenzylamine

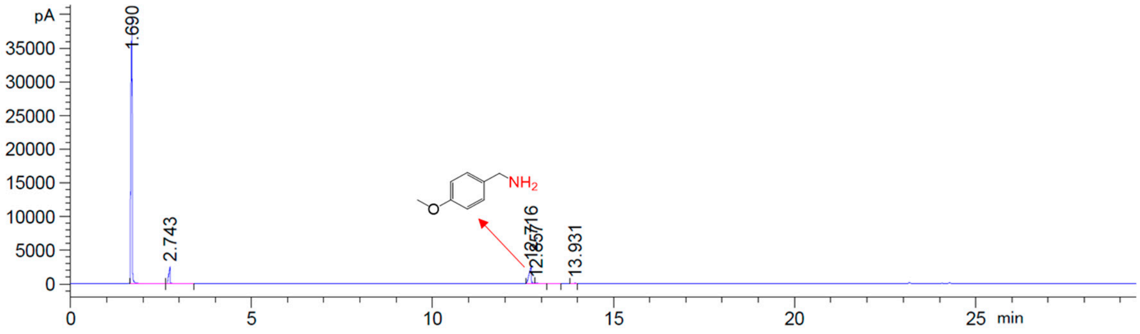

3,4-Dimethoxybenzylamine

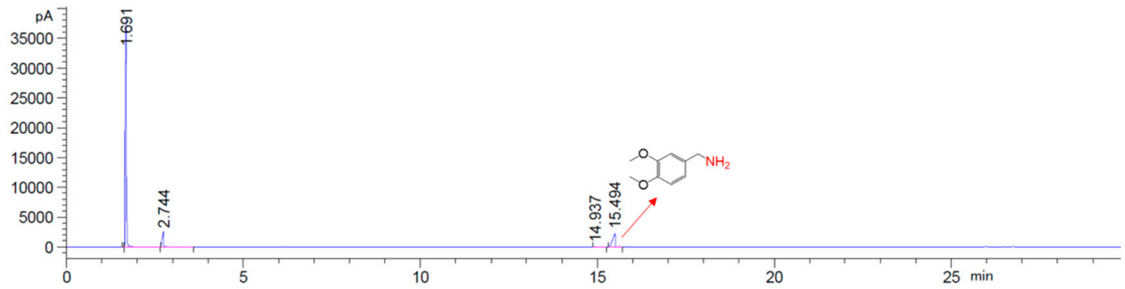

3-Phenylpropan-1-amine

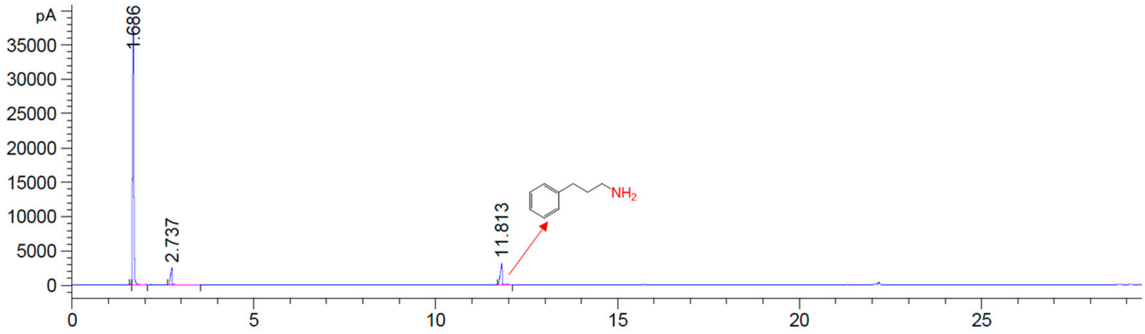

N-(4-(aminomethyl)phenyl)acetamide

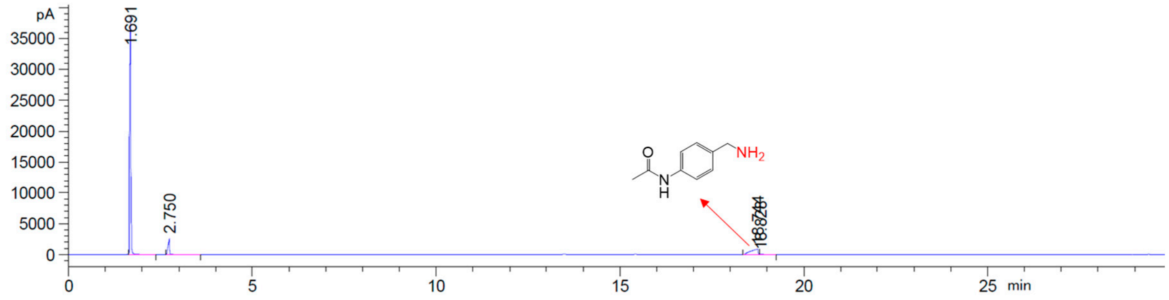

***n*-Butylamine**

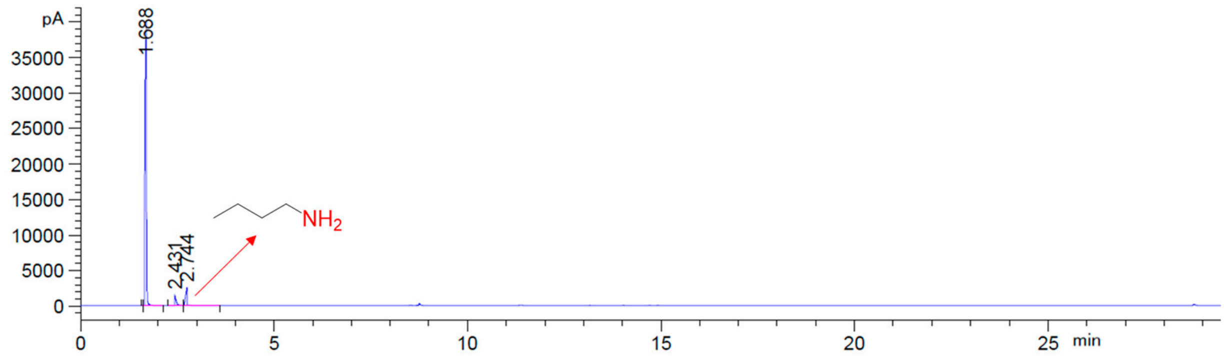

***n*-Amylamine**

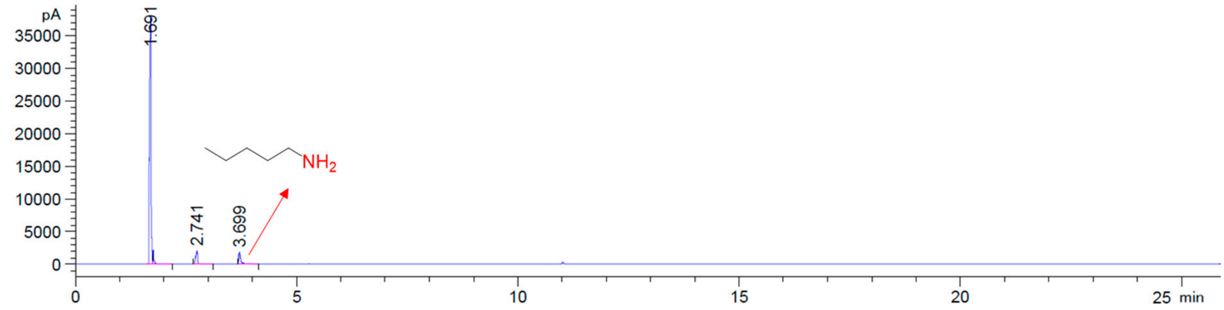

***n*-Hexylamine**

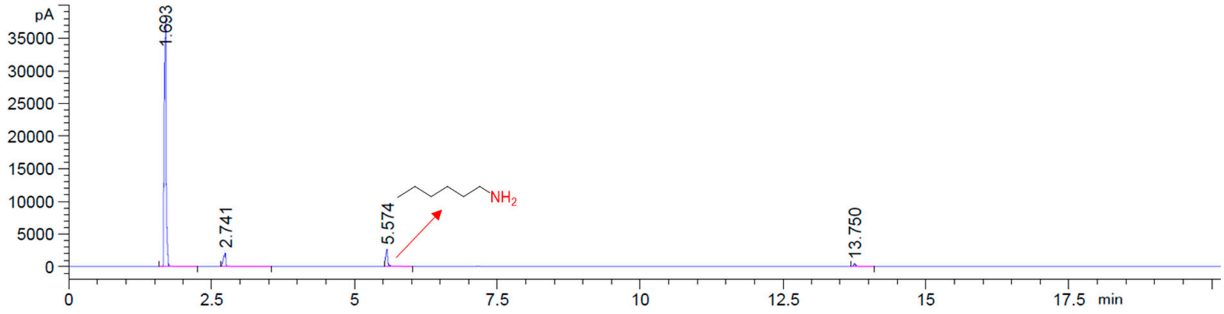

***n*-Octylamine**

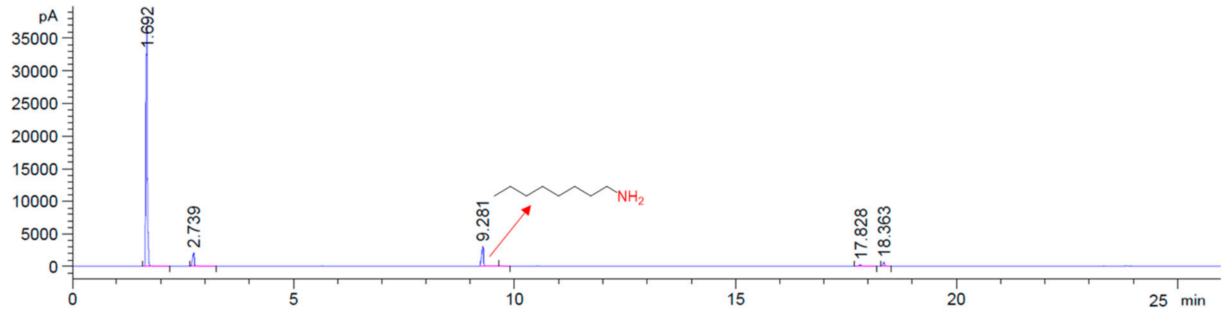

Furan-2-ylmethanamine

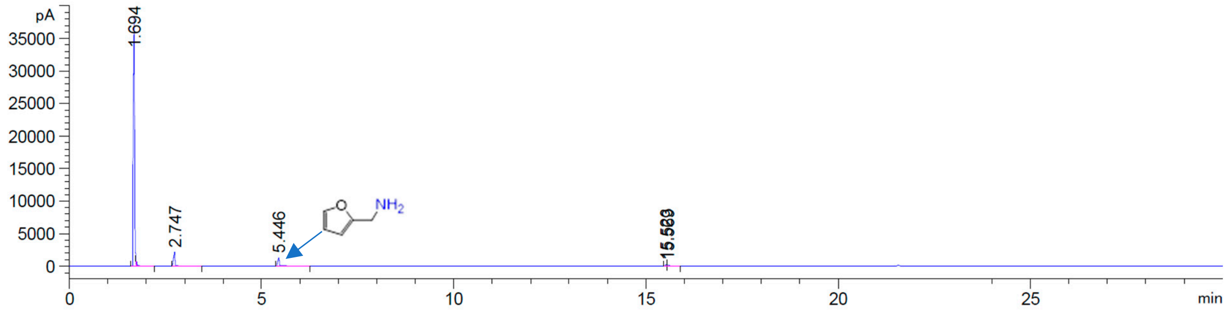

(5-(Aminomethyl)furan-2-yl)methanol

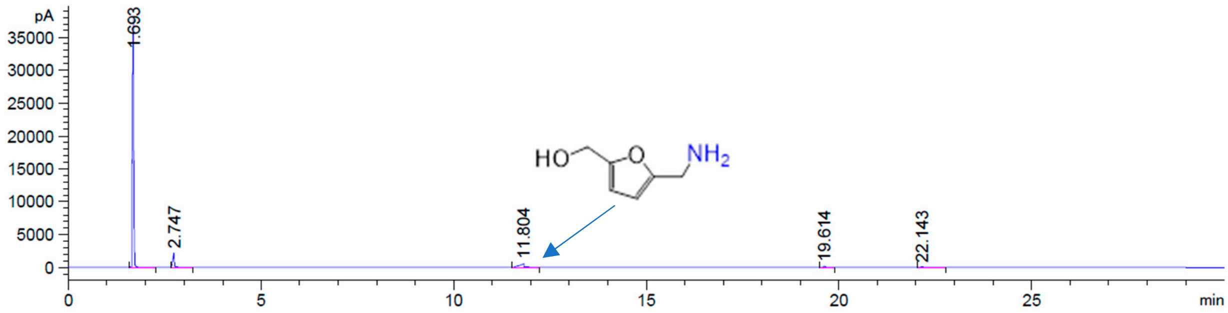

3,7-dimethyloct-6-en-1-amine

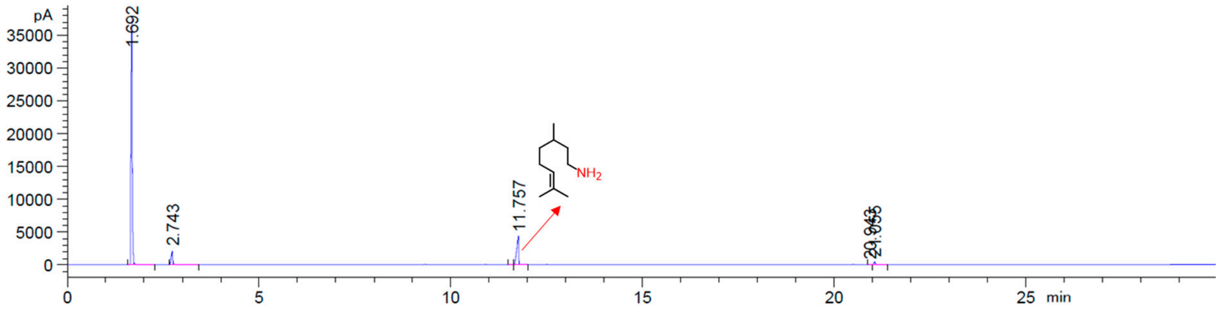

Supplement: Supplementary file 1 [file molecules-30-03089-s001.zip › molecules-3730780-supplementary.pdf]
